# Molecular identification of vivax malaria relapse patients in the Yunnan Province based on homology analysis of the *Plasmodium vivax* circumsporozoite protein gene

**DOI:** 10.1007/s00436-022-07700-7

**Published:** 2022-11-05

**Authors:** Yanchun Xu, Ying Dong, Yan Deng, Herong Huang, Mengni Chen, Yan Liu, Jing Wu, Canglin Zhang, Webi Zheng

**Affiliations:** 1 Yunnan Institute of Parasitic Diseases Control, Yunnan Provincial Key Laboratory of Vector-Borne Diseases Control and Research, Yunnan Centre of Malaria Research, Pu’er, 665000 China; 2 Department of Basic Medical Sciences, Clinical College of Anhui Medical University, Hefei, 230031 China; 3 Center for Disease Control and Prevention, Baoshan, 678000 China

**Keywords:** Yunnan, *Plasmodium vivax*, Relapse, Gene, Homology, Identification

## Abstract

More than 85% of the malaria burden in the Yunnan Province is caused by imported vivax malaria, and Yunnan is also where the majority of vivax malaria patients are diagnosed in China. Timely removal of the infection sources of *Plasmodium vivax* and its breeding environment remains the key to eliminating the secondary transmission of imported malaria. To that end, blood samples were collected from cases diagnosed and revalidated as single species infection with *P. vivax* in the Yunnan Province from 2013 to 2020. Specifically, samples from vivax malaria patients with suspected relapses episodes were subjected to PCR amplification, product sequencing, and analysis of the *P. vivax* circumsporozoite protein (*pvcsp*) gene. In total, 77 suspected relapse patients were identified out of 2484 cases infected with *P. vivax*, with a total of 81 recurrent episodes. A total of 156 CDS (coding DNA sequence) chains were obtained through PCR amplification and sequencing of the *pvcsp* gene from 159 blood samples, 121 of which can be matched to the paired sequences of 59 vivax malaria patients with both primary attack and recurrent experience. Of the 59 pairs of *pvcsp* gene sequences, every one of 31 pairs showed only one haplotype and no variant sites (VS), meaning every two paired sequence was completely homologous. Every one of the remaining 28 paired sequences had two haplotypes but no length polymorphism, indicating that the paired sequences was “weakly heterologous” with no fragment insertions (or deletions). All 59 vivax malaria patients with recurrences were caused by the activation of *P. vivax* hypnozoites originated from the same population as the primary infection. The paired analysis of the similarity between high variant genes allowed the identification of relapse episodes caused by *P. vivax* homologous hypnozoites and also demonstrated *pvcsp* gene as one of the candidate molecular markers for tracing infection origin.

**Supplementary Information:**

The online version contains supplementary material available at 10.1007/s00436-022-07700-7.

## Introduction

In recent years, as efforts to control malaria have increased, the proportion of *P. vivax* infections in many traditionally highly endemic areas, such as Sri Lanka, Thailand, and Brazil, has shown a counter-intuitive increasing trend. The cause may be related to the fact that previous prevention and control measures ignored the characteristics of *P. vivax* and indirectly contributed to the accumulation of *P. vivax* infection sources (Battle et al. 2015). It has been observed that mature gametophytes (stage V gametophytes) of *P. vivax* appear almost simultaneously with its asexual bodies in the early stages and continue to produce gametophytes throughout the infection period (Bockarie et al. 2006; Ghanchi et al. 2019). The high efficiency of transmission therefore also means that the most effective measures to remove the infection sources of *Plasmodium falciparum*, such as early diagnosis and timely treatment, may not be able to contain the danger of an early episode of *P. vivax* infection. In addition, *P. vivax* develops more rapidly inside *Anopheles* mosquitoes and can easily circumvent these interventions such as insecticide netting and indoor residual spraying. Thus, the maintenance of *P. vivax* populations is easier than those applicable to *P. falciparum* using the same vector control procedure (Golding et al. 2015). Moreover, due to the existence of a parasitic mechanism of hypnozoites, *P. vivax* can break through the local and seasonal limits of mosquito vector transmission (White et al. 2012; Battle et al. 2014). The accumulation of infection sources plays a negative role in increasing the complexity associated with *P. vivax* transmission*.*

The last indigenous malaria cases infected with *P. vivax* in China ceased in 2016. However, Yunnan remains the province with the largest number of imported vivax malaria cases, with instances primarily originating in Southeast Asian countries. To wit, in 2018, such cases accounted for 80.8% (172/213) of malaria cases caused by *P. vivax* infection throughout the province (Li et al. 2021), of which there was no shortage of groups with multiple episodes and suspected relapse of *P. vivax* infection (Huang et al. 2021). During the period spanning 2013–2019, suspected relapse events constituted approximately 3.5% (83 in total) of the 2364 vivax malaria patients diagnosed in the Yunnan Province. This was based on a minimum interval of 45 days between the first recurrence and the original episode (Llanos-Cuentas et al. 2014). Comparing other malaria endemic areas, this interval has an average duration of 41 days across those Southeast Asian countries where *P. vivax* is widely prevalent (Battle et al. 2014) and 7% of vivax malaria relapse within 6 months in Thailand (Llanos-Cuentas et al. 2014), suggesting the burden of vivax malaria relapse in Yunnan Province is no less than that of neighboring countries. Of course, other regions of the world are also facing the challenge posed by vivax malaria relapse, with the relapse rate in Papua New Guinea being as high as 80% (Betuela et al. 2012). Twenty-three percent of pregnant women in Brazil had a relapse episode due to failure to receive primaquine radical treatment (Corder et al. 2020); overcoming this negative result has become an unavoidable source of difficulty in the process towards the global elimination of malaria (Baird 2016; White et al. 2016). One of the three major technical bottlenecks in the control of vivax malaria is accurate identification of relapse infection caused by the activation of *P. vivax* hypnozoites (Baird 2016).

The WHO recommends analyzing the homology of single-copy antigenic genes of *P. vivax* as a method for the molecular identification of “new infection” and “recrudescence” events (Koepfli et al. 2009). Lin et al. (2015) identified 37.9% (11/29) of re-emergence vivax malaria episodes caused by the activation of *P. vivax* homologous hypnozoites based on analysis of the genetic similarity between paired strains from initial infection and the re-emergent strains. Craig et al. (1996), Imwong et al. (2012), and Chen et al. (2007) also assessed the differentiation characteristics of *pvama1* (*P. vivax* apical membrane antigen 1), *pvcsp*, *pvmsp1* (*P. vivax* merozoite surface protein 1), and other genes, which revealed the first vivax malaria relapse was mostly caused by the activation of *P. vivax* homologous hypnozoites. The feasibility of molecular identification of relapse events in *P. vivax* infection has been demonstrated from different perspectives. To establish a practical and reasonable method for evaluating the relapse episode of *P. vivax* infection, this study used nest polymerase chain reaction (PCR) for amplification of hypervariable genes (White et al. 2016), followed by DNA sequencing of PCR amplification product to identify vivax malaria recurrent events in the Yunnan Province. The results of the molecular characterization of the *pvcsp* gene and its marker role in identification of the relapse strains of *P. vivax* are reported below.

## Materials and methods

### Study subjects and blood samples

The study was conducted on cases from throughout Yunnan Province from January 2013 to December 2020 that were confirmed at the Yunnan Province Malaria Diagnosis Referent Laboratory (YPMDRL) using both examination by light microscopy of blood smears and genetic testing (SI 1) (Dong et al. 2020). All cases were officially registered and counted in the “China Disease Surveillance Information Reporting System,” from which suspected relapse individuals infected with *P. vivax* and with a history of re-emergence were further screened. The infection source of *P. vivax* was confirmed as indigenous or imported by epidemiological surveys at each outbreak of vivax malaria cases. The criteria are as follows: those with no history of movement outside Yunnan Province within 30 days prior to the onset of *P. vivax* infection were classified as indigenous cases in Yunnan Province, while those with a history of exposure to vivax malaria endemic areas such as Myanmar and Africa were classified as patients who imported Myanmar and African infections (SI 2). The following inclusion criteria were applied for the vivax malaria relapse: (1) patients who were clinically cured after an 8-day course of chloroquine/primaquine (in total 1550-mg chloroquine therapy within 3 days, followed by the subsequent 8-day course of 22.5-mg/day primaquine therapy) and had a second episode of *P. vivax* infection in the peripheral blood 28 days later; (2) no further history of exposure to malaria endemic areas was verified by epidemiological survey after the original episode. The data of recurrence from every vivax malaria patient are shown in SI 2.

Venous blood was collected from all vivax malaria patients during the primary infection and before treatment for recurrent episodes (day 0). The samples were prepared as filter paper dry blood drops for *Plasmodium* genetic analysis.

### Extraction of Plasmodium genomic DNA and PCR amplification of the pvcsp gene

Three filter paper dry blood drops with a diameter of 5 mm were used to extract *Plasmodium* genomic DNA. This was carried out in accordance with the instructions of the QIAgen Mini Kit (QIAamp, Germany), and the samples were subsequently stored at − 20 °C.

In the current study, PCR amplification products were sequenced to obtain the DNA sequence of the whole *pvcsp* gene, and the nested-PCR method, having relatively good sensitivity and specificity, was adopted in order to stably amplify the target gene from the extracted *Plasmodium* genome samples for each storage period. The reference sequence (ID: NC_009913.1) was used as the template to design the primers and reaction conditions for the nested PCR amplification of the *pvcsp* gene. The first round of PCR amplified the region of 1,537,513–1,539,033 that contains approximately 1521 base pairs (bp), with the upstream and downstream primers as 5′-CCGTTCGAACAAGTTCTGTTC-3′ and 5′-GCGCATAATGTGTAAGAGGTGT-3′, respectively. The second round amplified a region of 1341 or so bp from 1,537,625–1,538,965, for which the upstream and downstream primers were 5′-GCTTAAG TTAAGCAAGCAAAACAGC-3′ and 5′-GCAGGGAACATTCATAAGAAAGGG-3′, respectively. The system for both PCR reactions was the following: 2.6 μl of DNA template; 14 μl of PCR mixed with 2 × Taq; and 0.7 μl of each of the upstream and downstream primers (10 umol/L), then ddH_2_O was added to reach a volume of 25 μl. It was conducted under the following sets of PCR reaction conditions: 94 °C for 3 m; 94 °C for 30 s, 49 °C for 90 s, 72 °C for 2 m, 35 cycles; or 72 °C for 7 m. The amplified products after second round PCR were carried out using 1.5% agarose gel electrophoresis for result observation, then the amplified products were sent to Shanghai Meiji Biomedical Technology Co. Ltd. for Sanger sequencing.

### Gene polymorphism and homology analysis of pvcsp genes in paired blood samples

The sequencing results were collated in DNASTAR 11.0 and BioEdit 7.2.5. The obtained DNA sequences were compared with the reference sequences (XM_001616843.1) of the *pvcsp* gene. When the “query cover” and “identifies” were greater than 98%, it indicated that the sequences collated were the intended targets. DnaSP 6.11.01 software was used to confirm the locus mutations and haplotypes of the *pvcsp* gene, from which the expected heterozygosity (He) and nucleic acid diversity index (π) of the DNA sequences were calculated. The *pvcsp* gene sequences of paired primary and relapse isolates from the same individual patients with several episodes of *P. vivax* infection were identified (Craig et al. 1996; Chen et al. 2007) separately using MEGA 5.04 software for similarity matching and central repetitive region (CRR) analysis (Imwong et al. 2005; Coppi et al. 2011; Dias et al. 2013). These amino acid residue regions including N-terminal, KLKQP five amino acids, and the C-terminal deduced from the *pvcsp* gene sequence were named *α*-N, region I (RI), and thrombospondin repeat (TSR), respectively (Coppi et al. 2011). When the main peptide repeat motifs (PRMs) of the CRR were “GDRA[D/A]GQPA” and “ANGAGNQPG,” they were identified as VK210 and VK247 type sequences, respectively (Rosenberg et al. 1989; Imwong et al. 2005; Dias et al. 2013), while “APGANQ[E/G]GAA” was confirmed as the PRM of the *P. simiovale*-type sequences (Qari et al. 1993).

### Confirmation of the paired P. vivax strains and the nature of the recurrent episode

When the CRR repeat motifs deduced from two DNA sequence in each paired samples collected from the same vivax malaria patient showed the following characteristics, the *P. vivax* strains from both the original infection and the recurrent episodes were considered to be inoculated by the same mosquito, and the subsequent vivax malaria attacks in this patient were considered to be caused by the activation of *P. vivax* hypnozoites in this population. Then this kind of recurrent episodes were termed as relapse episodes (Imwong et al. 2007): (1) only one haplotype was shown, and both the expected heterozygosity (He) and quantity of VS were equal to 0. This indicated the *P. vivax* strains from paired samples were completely homologous single-clone strains (Khusmith et al. 1998; Chen et al. 2007; Koepfli et al. 2009; Imwong et al. 2012); (2) the number of haplotypes was greater than one, and both He and VS greater than 0. VS was confirmed by checking peak plots (SI 3), from which it was seen that the paired DNA sequences were of the same length and, with no fragment deletions (or insertions), suggesting the paired *P. vivax* strains were sibling strains and that *pvcsp* genes were weakly heterologous but did not undergo intra-helical recombination events (Brito et al. 2011; Dias et al. 2013).

## Results

### Sample information and PCR amplification product sequencing

In total, 77 reports (3.1%) with recurrent episodes were retrieved from 2484 cases infected with *P. vivax*, drawn from the period between 2013–2019, all of which patients were living in Yunnan Province (97°31′ E to 106°11′ E; 21°8′ N to 29°15′ N). The majority of patients could be traced to origins in Myanmar (98.7%, 76/77), and the male-to-female ratio was 5 males per 1 female for all patients in the study. The majority of patients had a single relapse (97.4%, 75/77), while one patient had episodes twice, and another patient had three episodes. General demographic information and original place of infection for the 77 patients are shown in Table 1 (SI 2).

**Table 1:** Demographic and clinical characteristics of the study cohort

Variable	Total	2013	2014	2015	2016	2017	2018	2019	2020
Total	77	0	14	19	17	9	9	7	2
1. Gender									
Male	64	0	12	16	13	7	9	5	2
Female	13	0	2	3	4	2	0	2	0
2. Age (in years)									
0–20	8	0	1	0	4	3	0	0	0
21–60	66	0	12	19	13	5	9	6	2
Above 60	3	0	1	0	0	1	0	1	0
3. Malaria recurrence									
1 episode	75	0	13	19	17	9	8	7	2
2 episodes	1	0	0	0	0	0	1	0	0
3 episodes	1	0	1	0	0	0	0	0	0
4. Infection source									
Myanmar	76	0	13	19	17	9	9	7	2
Africa	1	0	1	0	0	0	0	0	0
Yunnan indigenous	0	0	0	0	0	0	0	0	0
5. Interval time of recurrence (days)									
Longest	1561	0	939	882	1561	426	319	367	268
Shortest	28	0	54	46	62	80	43	28	264
Average	279	0	271	291	292	277	275	285	264

Data are no. (%) of paired samples, unless otherwise indicated

**Fig. 1:**
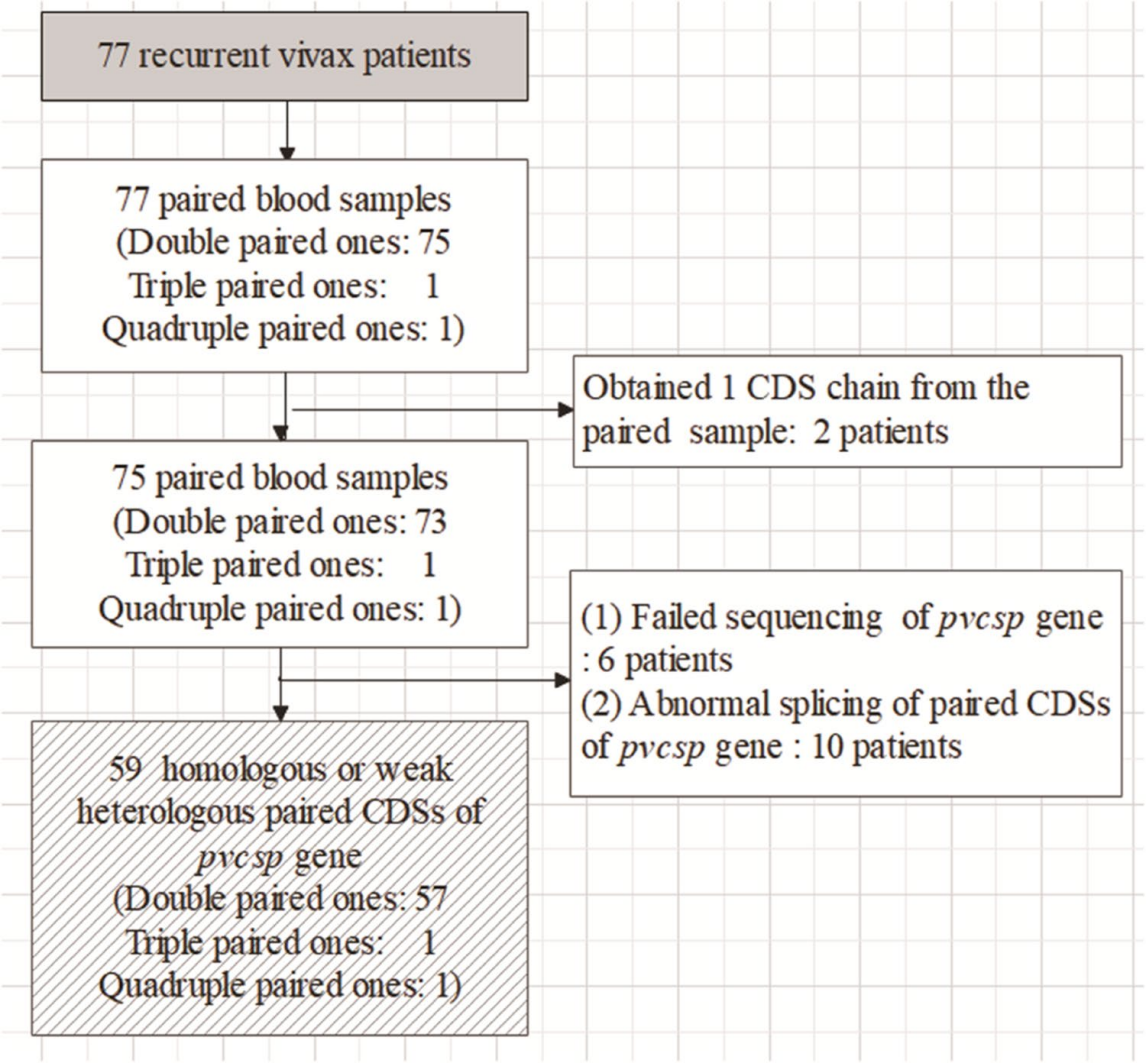
Paired *pvcsp* gene sequencing results obtained from relapse patients infected with *P. vivax*, in Yunnan Province from 2013 to 2020

A total of 81 recurrent episodes occurred among these 77 patients infected with *P. vivax*, allowing a total of 159 blood samples to be obtained from all reported original infection and recurrent episodes. From these, 156 PCR amplification products of the *pvcsp* gene were successfully obtained, which showed strong signal target bands longer than 1000 bp and shorter than 1500 bp in the electrophoregram (SI 4), with a product acquisition rate of 98.1% (156/159). Of them, paired CDS full strands (807–1179 bp in length) of the *pvcsp* gene were obtained from blood samples in 75 patients (97.4%, 75/77), but only those from 59 patients could be used for homology analysis of the gene sequences (Fig. 1).

The structural regions of the amino acid chains derived from the CDS strand conversions were completed, including the conserved region near the 5′-end (coding 1st–90th aa of PvCSP) (*α*-N region), the R I (KLKQP) encoding region, near the 3′-end (coding 276th–393th aa of PvCSP) (TSR) and the highly variable region in *pvcsp* middle for encoding CRR of PvCSP (96th–275th aa), etc. (SI 5).

### Diversity of the pvcsp gene and the CRR array of PvCSP

The 121 CDS strands (GenBank ID: OP354287–OP354399) of *pvcsp* gene obtained from paired blood samples of 59 patients showed 32 double alleles at positions 112, 113, 233, 234, 240, 261, 264, 270, 274, 282, 295, 309, 327, 347, 354, 426, 491, 507, 511, 534, 545, 552, 572, 579, 615, 684, 742, 761, 769, 805, 892, and 999 (bimodal chart), with a nucleotide diversity index (π) equal to 0.1384 (± 0.0056). The sequences from the original infection and recurrent strains both call only one of the biallelic bases, usually the type with a strong sequencing signal (SI 3). The 32 double alleles were distributed in all seats of the *pvcsp* gene but were predominantly concentrated in the CRR region (62.5%, 20/32) (Table 2).

**Table 2:** The polymorphism of single nucleotide loci in pvcsp gene CDS chains

Regions	Loci	Alleles of call （major/minor^a^）	Coding	Amino acid variation	No. of CDS （*n*=121）	Frequency
*α*-N^b^	c.112	A/G	AAC/GGC	N38G	2	0.0165
	c.113	A/G			2	0.0165
	c.233	A/G	GAG/GGG	E78G	2	0.0165
	c.234	G/T	GAG/GAT	E78D	20	0.1652
	c.240	A/G	AAA/AAG	K80K	18	0.1488
	c.261	A/C	CCA/CCC	P87P	14	0.1157
	c.264	T/G	CGT/CGG	R88R	4	0.0331
RI^c^(91th–95th aa)	c.274	T/C	TTG/CTG	L92L	16	0.1322
	c.282	A/G	CAA/CAG	Q94Q	2	0.0157
CRR (96th–290th aa)	c.295	C/A	CGA/AGA	R99R	2	0.0165
	c.309	G/A	CAG/CAA	Q103Q	2	0.0165
	c.327	A/C	GGA/GGC	G109G	2	0.0165
	c.354	A/C	GGA/GGC	G118G	4	0.0315
	c.426	C/A	GGC/GGA	G142G	2	0.0165
	c.491	G/C	GGT/GCT	G164A	2	0.0165
	c.507	A/T	GGA/GGT	G169G	2	0.0165
	c.511	G/A	GGA/AGA	G171R	2	0.0165
	c.518	C/A	GCT/GAT	A173D	2	0.0165
	c.534	C/A	GGC/GGA	G178G	2	0.0165
	c.545	A/C	GAT/GCT	D182A	2	0.0165
	c.552	T/A	CAT/CAA	Q184R	2	0.0165
	c.572	G/C	AGG/AGC	R286S	2	0.0165
	c.579	G/A	CAG/CAA	Q193Q	2	0.0165
	c.615	A/C	GGA/GGC	G205G	2	0.0165
	c.684	A/C	GGA/GGC	G228G	6	0.0496
	c.742	C/G	CCA/GCA	P248A	2	0.0165
	c.761	C/G	AGC/AGG	G254R	2	0.0165
	c.769	C/G	CCA/GCA	P257A	2	0.0165
	c.805	A/C	ACC/CCC	P269T	2	0.0165
TSR^d^(291th–393th aa)	c.892	C/T	CTT/TTT	L298F	2	0.0165
	c.999	A/G	AAA/AAG	K333K	2	0.0165

a: At the double allelic base in the DNA sequencing peak map, the base with higher wave peak is major allele, and the other base with lower wave peak is minor allele; b named the near N-terminal of PvCSP amino acid chain; c the coding region of KLKQP five amino acids; d: the C-terminal of PvCSP amino acid chain

Furthermore, 56.3% (18/32) of the polymorphic sites belonged to the third base of the amino acid codon, and only 27.8% (5/18) of these resulted in amino acid variants. The proportion of the second base and first base were 17.6% (6/34) and 26.5% (9/34), respectively (Table 2), while the highest frequency of the double allele was 0.165 for c.234, the minor allele frequency (MAF) was 0.1488 for c.240, and 75.0% (24/32) of the double alleles were present in only one set of paired sequences (Table 2).

**Fig. 2:**
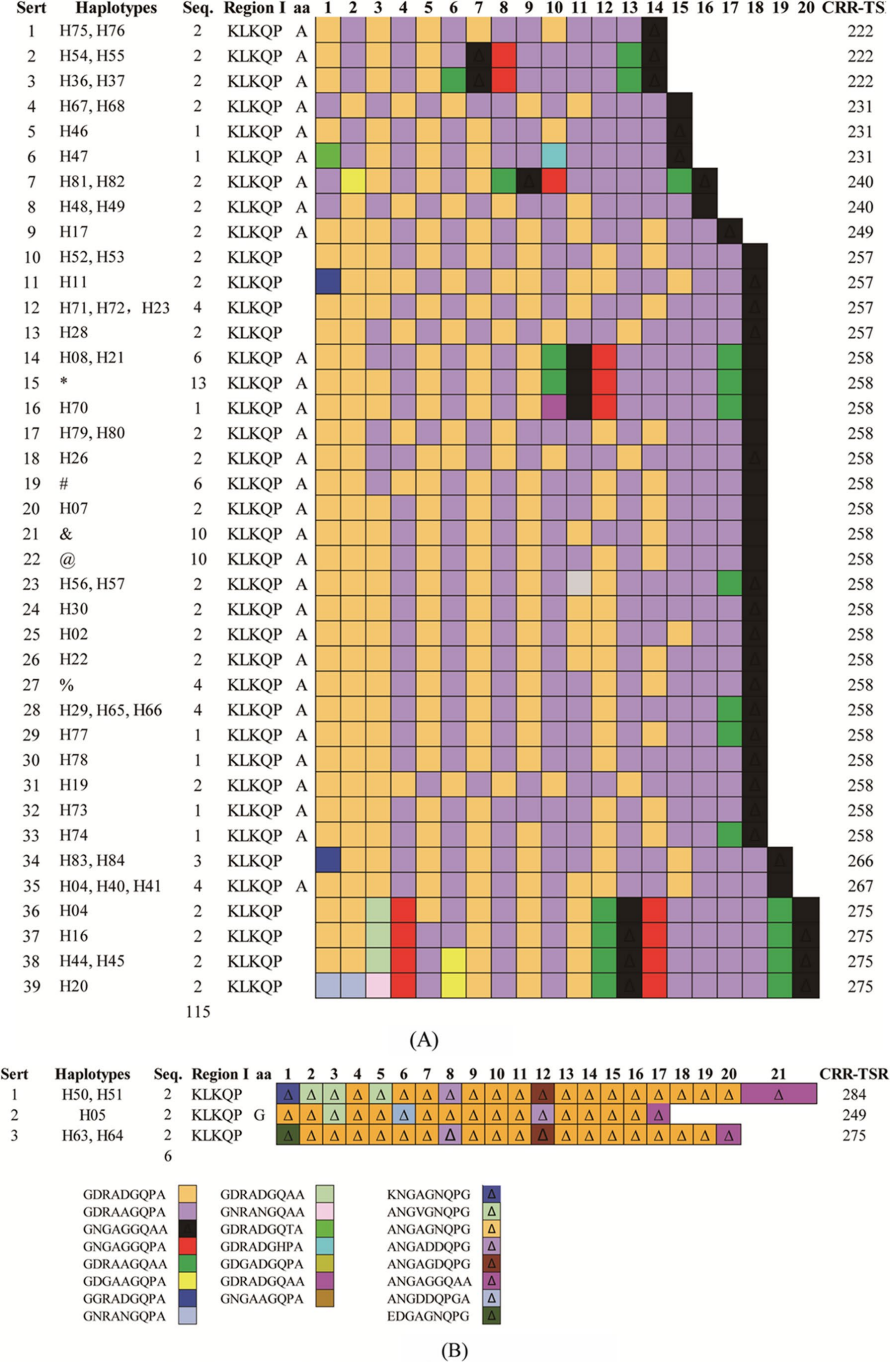
CRR repeats of PvCSP deduced from each haplotype of the *pvcsp* gene in *P. vivax* strains (**A** VK210; **B** VK247) made in Excel. (1) *: including H09, H10, H25, H42, H43, H13, H35, and H69. (2) #: Including H31, H32, H33, H34, and H15. (3) &: Including H01, H12, H14, H27, H58, and H59. (4) @: Including H03, H18, H61, H62, and H24. %: Including H24, H60, H38, and H39

In addition to the aforementioned, 121 CDS strands were defined as 84 haplotypes (H01 to H84) with a He of 0.9940 (± 0.0040). Of these, haplotypes H05, H50, H51, H63, and H64 had CRR repeat units (PRMs) of the VK247 genotype (Fig. 2B), while the remaining 79 had the PRMs characteristic of the VK210 genotype (Fig. 2A).

Among the haplotypes of the VK210 type, there were 39 CRR forms consisting of peptide repeat motifs (PRMs) (Fig. 2A). Of these, there were 15 PRM unit types, with GDRAAGQPA and GDRADGQPA occurring most often, with frequencies of 0.470 (987/2100) and 0.3833 (805/2100), respectively. The remaining 13 PRMs, included the five newly detected PRMs GNRANGQPA (0.0033, 7/2100), GNRANGQAA (0.0001, 1/2100), GDRADGQTA (0.0001, 1/2100), GDRADGHPA (0.0001, 1/2100), and GNGAAGQPA (0.0001, 1/2100) (Fig. 2A). Generally, the CRRs of VK210 type consisted of 14–20 PRMs with 18 being the most common, and 96.8% (38/39) ended with GNGAGGQAA units (Fig. 2A). The CRRs of the paired sample from imported patient 24 infected in Africa (SI 1) were defined as Hap-23 of VK210 type, which did not show any divergent whit Myanmar strains (Fig. 2A).

Of the five haplotypes of type VK247, three types of CRR consisted of 17–21 PRMs (Fig. 2B) in which there were eight unit types of PRMs. Those with the highest frequency of occurrence were ANGAGNQPG (0.7414, 86/116), ANGAGGQAA (0.0517, 6/116), and ANGDDQPGA (0.0172, 2/116), and the remaining two were newly detected PRMs (Fig. 2B).

### Comparison of paired blood samples of the pvcsp gene and confirmation of relapse episodes

The results of alignment of the paired *pvcsp* gene CDS chains of the 59 paired blood samples showed that every paired CDS chains of 31 groups (52.5%, 31/59) had only one haplotype and no VS, and the He and VS values were both 0. This indicated that each of the 31 pairs was homologous and the source of the paired *P. vivax* strains was a single clone with complete genetic homology, belonging to the same mosquito bite–inoculated population (Table 3). Subsequent episodes of *P. vivax* infection were caused by the activation of hypnozoites from the same population as primary infection strains. Every paired CDS chains from the other 28 paired blood samples (47.5%) had varying numbers of polymorphic sites (1–6 loci) between each pair. However, there were two exceptions, at c.39 (0.0082, 1/121) and c.1027 (0.0082, 1/121), which were true base substitutions (Table 3, SI 3), while the remaining sites were all double allelic bases (Table 2). These 28 paired sequences did not show the trace of DNA fragment insertion (or deletion), suggesting they were heterologous with few base substitutions, but did not experience intra-helical recombination events. Also, their heterogeneity resulted from sibling strains that were not completely homologous genetically and belonged to the latter generations bred from the common ancestor inoculated at one time by mosquito bite (Table 3). Subsequent recurrent episodes of *P. vivax* infection were still caused by the activation of hypnozoites in the same population as primary infection strains.

**Table 3:** Identification of the homology of *P. vivax* strains from each group paired sample based on alignment of the *pvcsp* gene sequence

No. of paired samples	Length of aa chains	Nucleotide diversity (Pi)	Ins or Del in CRR (bp)	Combined variable (polymorphic) sites					Paired strains	Single infection
				No.	*α*-N(1^th^-90^th^ aa)	RI (91^th^-95^th^ aa)	CRR (96^th^-290^th^aa)	TSP (291^th^-393^th^ aa)		
I. Paired (groups=59)										
31	364-393	0	0	0	0	0	0	0	com-Homo	Yes
8	340-376	0.0009	0	1^a^	234, 240, 261, 264	274	295	0	non-re-Sibl	Yes
9	340-376	0.0018	0	2^b^	234, 240, 261, 270	274	327, 354, 507, 552, 572, 615, 742	999, 1027^*^	non-re-Sibl	Yes
4	333-386	0.0030	0	3^c^	234, 240, 261, 264	274, 282	491, 511, 545	0	non-re-Sibl	Yes
3	372-376	0.0036	0	4^d^	234, 240, 261	274	426, 518, 534, 742, 761, 769	0	non-re-Sibl	Yes
3	336-375	0.0049	0	5^e^	112, 113, 233, 234, 240, 261, 270	274	309, 579, 684, 805	892	non-re-Sibl	Yes
1	358-368	0.0054	0	6^f^	39^*^, 234, 240, 261, 270	274	0	0	non-re-Sibl	Yes
II. Any tow CDSs of non-paired samples										
69 and 109^**^	372 and 368	0.0263	+12^g^	29^f^	240, 247^*^, 259^*^, 264	0	291^*^,294^*^,318^*^,321^*^,345^*^, 348^*^,363^*^,444^*^,453^*^,552, 561^*^,572,579,588^*^,599^*^, 606^*^,615,642^*^,783^*^,798^*^, 800^*^,803^*^,805	29^f^	dif-Popu	No
156 and 110^##^	393 and 368	0.0245	+54^g^	27^f^	261	0	291^**^,294^*^,318^*^,321^*^,345, 348^*^,363^*^,364^*^,372^*^,373^*^, 376^*^,390^*^,444^*^,453^*^,552, 561^*^,572,579,588^*^,599^*^,669^*^,687^*^,696^*^,707^*^,714^*^,796^*^	27^f^	dif-Popu	No
49 and 125^&&^	275 and 249	0.0204	+(51+27)^h^	22^f^	240, 261, 264	274	404^*^,435^*^,567^*^,570^*^,591^*^, 594^*^,597^*^,598^*^,621^*^,645^*^, 648^*^,650^*^,651^*^,652^*^,702^*^, 705^*^,729^*^,780^*^	22^f^	dif-Popu	No

a–e：Including one locus polymorphic and any two, three, four, five loci polymorphic. f: Including all loci polymorphics at the same time. g: “+” Including one inserted peptide composed of many amino acids. h: Including two inserted peptides composed of many amino acids. *：Not showing the double peaks at the base site in sequencing peak map. **: Alignment of the two CDS chains of the original infected strains of both patient1 and patient3. ##: Alignment of the CDS chain of original infected strains from patient4 with the CDS chain of relapse infected strains patient3. &&: Alignment of the two CDS chains of the original infected strains of both patient53 and patient5

However, similarity matching of two randomly sequences from the 121 CDS strands showed there were significantly more base substitutions between the two sequences. Furthermore, length polymorphism presented between the two unpaired CDS strands due to the presence of different length oligonucleotide strand insertions (or deletions) (Table 3). This indicated that the two sequences of the unpaired samples were more heterologous and their corresponding genomic donors, *P. vivax*, were not more likely to belong to the same population inoculated by the same mosquito (Table 3).

## Discussion

The single-copy *pvcsp* gene commences at the tip of *P. vivax* chromosome 8 (Brito et al. 2011; Dias et al. 2013; NCBI 2022), extends for 807–1179 bp, and does not possess introns but has a CDS that encodes polypeptides between 269 and 393 aa long. The structural diversity of the *pvcsp* gene is concentrated in the mid-segment encoding CRR repeat region from the 90th to 275th aa and its flanks (Fig. 1) (Imwong et al. 2005; Dias et al. 2013; Võ et al. 2020). It is generally characterized by insertions and deletions of repetitive units, mostly due to sexual recombination during meiosis or intra-helical strand-slippage events during mitotic DNA replication (McConkey et al. 1990; Kim et al. 2006; Bousema et al. 2011), as well as frequent base substitutions within the locus. Using the repeat units of the CRR as markers, *P. vivax* strains can be defined as three genotypes: VK210; VK247; and the *P. simiovale*, which is *P. vivax*-like (Qari et al. 1993; Tripathi et al. 2011). Therefore, *pvcsp* gene is commonly used as a molecular marker for the evolution of population genetic differentiation of *P. vivax* (di Giovanni et al. 1990; Brito et al. 2011; Kosaisavee et al. 2011; Etemadi et al. 2020).

This study provides preliminary pathogenic evidence on vivax malaria relapse episodes based on the analysis of *pvcsp* gene differentiation in the sample group which occurred in Yunnan Province. Although the *P. vivax* population included in this study was not truly a natural population, 97.4% of the strains in the sample were nonetheless harvested from patients infected in Myanmar. Consequently, the *pvcsp* genes of the entire sample group showed similar findings to those reported by Thanapongpichat et al. (2013) and Võ et al. (2020), which related to the Myanmarese population. These included the CRR region, the same significant polymorphism in length and composition of PRMs, the same predominance of VK210 types (95.0%, 115/121), and the absence of *P. vivax*-like variants. Furthermore, there was the same extremely low DNA sequence homology, with up to 84 haplotypes in 121 *pvcsp* gene sequences, though the expected heterozygosity (He, 0.9940 ± 0.0040) was greater than that observed by Võ et al. (He, 0.096 ± 0.034) (2020). However, at the same time, the RI of the *pvcsp* gene in this set of samples had only one type of arrangement of KLKOP, far fewer than the seven types described by Võ et al. (2020). In addition to this, there was a reduction in variety of PRMs units constituting the CRR region (VK210: 15, VK247: 8) and less complex arrangements and length polymorphisms than those reported in the aforementioned research. These may be justified by this study having involved a relatively homogeneous population of primarily Myanmarese strains of *P. vivax*, unlike the wider range and a more complex population composition of samples taken by Võ et al. However, the displayed high variability of the *pvcsp* gene was largely consistent with the results obtained in the current study. Furthermore, it is agreed that the CRR is suitable as a molecular marker for separating genetic differences in *P. vivax* populations due to its poor conservation, whereas the *α*-N and TSR regions on both flanks are suitable as candidate antigen genes for polyclonal antibody serum preparation because of their highly conserved sequences (Coppi et al. 2011; Dias et al. 2013).

In addition to confirming the high degree of polymorphism in the *pvcsp* gene structure at the population level, the similarity comparison performed with paired blood samples from the vivax malaria recurrent patients (*n* = 59) showed that the *P. vivax* strains with nearly identical gene sequences were always present rather than an accidence. In 52.5% (31/59) of the patients, the paired *pvcsp* gene sequences of *P. vivax* strains from the original infection and relapse were not only highly consistent in length but also completely similar in sequence structure, with both He and VS equal to 0, indicating complete homology of the paired sequences. Even among the 28 sets of paired sequences with double allelic expression (Tables 2 and 3), the *pvcsp* gene sequences were not only highly concordant in length. Conversely, there were notable oligonucleotide fragment insertions (or deletions) in any two of the *pvcsp* gene sequences from non-paired blood samples and there was also a significant increase in the number of base substitutions (Table 3). It is suggested that the length polymorphism in the *pvcsp* gene may be an important point of differentiation between populations of *P. vivax*, compared to the degree of single nucleotide polymorphism.

Besides the possible causes of reinfection and recrudescence (De Niz et al. 2018; Brito et al. 2022),

it is necessary to validate whether the activation of the intrahepatic *P. vivax* hypnozoites result in the vivax malaria frequent recurrence after antimalaria treatment (Looareesuwan et al. 1987; Imwong et al. 2007; Orjuela-Sánchez et al. 2009). In the 77 vivax malaria patients with recurrent episodes included in this study, history of exposure in malaria endemic areas outside the country occurred before the original infection episode only. Therefore, the possibility of recurrence caused by reinfection is basically excluded. The molecular identification of relapse in only 59 (76.6%, 59/77) patients was conducted by alignment of paired *pvcsp* gene sequences. In 31 of these patients, including those with two or three relapses, the paired *pvcsp* gene sequences of *P. vivax* strains from the original infection and the relapse episode were identical in length and had no VS within the sequences. This observation suggested that single clone strains of a genetically identical *P. vivax* existed in every paired blood samples, and the vivax malaria relapse could be attributed to the activation of intrahepatic hypnozoites with identical gene structure to the original infection strain (Lin et al. 2015; Craig et al. 1996; Chen et al. 2007; Imwong et al. 2007; Kirchgatter et al. 1998). In the other 28 patients, there was still a large degree of reproducibility in gene structure between the paired *pvcsp* gene sequences, which were identical in length, and the single nucleotide polymorphic sites were also mostly double alleles (Tables 2 and 3). Such an appearance of locus polymorphism may have been due to differential calling of double allelic bases in paired sequences by first generation sequencing (Orjuela-Sánchez et al. 2009), suggesting the sibling strains (Brito et al. 2011; Dias et al. 2013; Bright et al. 2014) with identical genetic structure existed in every group of paired blood samples, and the two batches of *P. vivax* populations from primary infection and relapse actually reflect parallel homology of polyclonal strains. Therefore, the authors suggested that the relapse of these 28 patients infected with *P. vivax* still belonged to the activation event of intrahepatic hypnozoites coming from common ancestor strains inoculated by a single mosquito bite occurrence (Conway et al. 1991; Sutton et al. 2009; Lin et al. 2015; Bright et al. 2014). It is worth examining whether the polymorphism form resulting from the differential mobilization of the double alleles is equivalent to the previously mentioned “weakly heterologous” genes (Chan et al. 2012; Nair et al. 2014; Friedrich et al. 2016; Popovici et al. 2018).

In summary, this paper analyzes the possibility that relapse episodes caused by homologous hypnozoites could be identified by finding the strong similarity between highly variable genes in *P. vivax*. Thus, methodological implications are provided for the identification of relapse episodes in malaria-free transmission areas, although there are some shortcomings. First, the study may still be incomplete in excluding recrudescence events caused by chloroquine-resistant strains or continued proliferation of myelo-plasmodium (Obaldia et al. 2018; Silva-Filho et al. 2020) due to the lack of feasible experimental verification means, although the chloroquine resistance associated molecular marker has been detected in some *P. vivax* strains from individual paired samples (SI 6). Secondly, the relapse event was confirmed by the similarity of *P. vivax* genes between the paired samples from the original infection and the reactivation. It included two results of 100% homology and “weakly heterologous” with only a few base substitutions between paired gene sequences, and it remains to be confirmed whether or not the latter was part of the activation of heterologous hypnozoites populations that scholars have previously studied. Thirdly, no definitive confirmation could be provided concerning the nature of the paired *P. vivax* strains in 23.3% (18/77) of patients. Although the authors are continuing to mitigate the limitations of single genetic markers to identify homologous strains, as well as in other investigations of these relapse causes, the length of this article does not allow for further elaboration, which may somewhat reduce the apparent completeness of this paper.

## Conclusion

In conclusion, this study identified vivax malaria relapse episodes caused by homologous hypnozoites using deep sequencing and analyzing the similarities of *pvcsp* genes in paired *P. vivax* strains from primary attack and relapse. The *pvcsp* gene is suitable as a candidate molecular marker to identify *P. vivax* homologous strains due to being simple in structure and easy to find in the traces of recombined gene. This study also demonstrated that most of vivax malaria relapse patients reported in Yunnan Province resulted from the single clone strains or sibling strains homologous with the original *P. vivax* infection. It is suggested that alignment of the gene similarity between paired *P. vivax* strains is suitable for identifying homologous hypnozoites activation.

## Supplementary Information

Below is the link to the electronic supplementary material.

Supplementary file1 (DOC 365 KB)

Supplementary file2 (DOC 176 KB)

Supplementary file3 (DOC 2539 KB)

Supplementary file4 (DOC 603 KB)

Supplementary file5 (DOC 69169 KB)

Supplementary file6 (DOC 275 KB)

## Data Availability

The datasets used and/or analyzed during the current study are available from the corresponding author upon reasonable request. Nucleotide sequence data reported in this paper are available in GenBank under accession numbers OP354287 to OP354399.

## Abbreviations

*pvcsp*: *Plasmodium vivax* Circumsporozoite protein gene
P. vivax: Plasmodium vivax
P. falciparum: Plasmodium falciparum
CDS: coding DNA sequence
pvama1: P. vivax apical membrane antigen 1
pvmsp1: P. vivax merozoite surface protein 1
PCR: polymerase chain reaction
YPMDRL: Yunnan Province Malaria Diagnosis Referent Laboratory
bp: base pairs
He: expected heterozygosity
π: nucleic acid diversity index
PvCSP: P. vivax circumsporozoite protein
CRR: central repetitive region
PRMs: peptide repeat motifs
α-N: N-terminal of PvCSP
RI: region I, codons region of KLKQP five amino acids
TSR: thrombospondin repeat
VS: variable sites
MAF: minor allele frequency
Com-Homo: completely Homologous
non-re-Sibl: non-recombinated sibling
dif-Popu: different population
pvcrt-o: Plasmodium vivax chloroquine resistance protein ortholog

## References

[CR1] Baird JK (2010). Eliminating malaria—all of them. Lancet.

[CR2] Baird JK (2016). Attacking *Plasmodium*
*vivax*. Am J Trop Med Hyg.

[CR3] Battle KE (2014). Geographical variation in *Plasmodium*
*vivax* relapse. Malar J.

[CR4] Battle KE (2015). Global database of matched *Plasmodium*
*falciparum* and *P. vivax* incidence and prevalence records from 1985–2013. Sci Data.

[CR5] Betuela I (2012). Relapses contribute significantly to the risk of *Plasmodium*
*vivax* infection and disease in Papua New Guinean children 1–5 years of age. J Infect Dis.

[CR6] Bockarie MJ (2006). Are insecticide-treated bednets more protective against *Plasmodium*
*falciparum* than *Plasmodium*
*vivax*-infected mosquitoes?. Malar J.

[CR7] Bousema T (2011). Adjusting for heterogeneity of malaria transmission in longitudinal studies. J Infect Dis.

[CR8] Bright AT (2014). A high resolution case study of a patient with recurrent *Plasmodium*
*vivax* infections shows that relapses were caused by meiotic siblings. PLoS Negl Trop.

[CR9] Brito CF (2011). Molecular markers and genetic diversity of *Plasmodium*
*vivax*. Mem Inst Oswaldo Cruz.

[CR10] Brito MAM (2022). Morphological and transcriptional changes in human bone marrow during natural *Plasmodium*
*vivax* malaria infections. J Infect Dis.

[CR11] Chan ER (2012). Whole genome sequencing of field isolates provides robust characterization of genetic diversity in *Plasmodium*
*vivax*. PLoS Negl Trop Dis.

[CR12] Chen N (2007). Relapses of *Plasmodium*
*vivax* infection result from clonal hypnozoites activated at predetermined intervals. J Infect Dis.

[CR13] Conway DJ (1991). The epidemiology of multiple-clone *Plasmodium*
*falciparum* infections in Gambian patients. Parasitology.

[CR14] Coppi A (2011). The malaria circumsporozoite protein has two functional domains, each with distinct roles as sporozoites journey from mosquito to mammalian host. J Exp Med.

[CR15] Corder RM (2020). Quantifying and preventing *Plasmodium*
*vivax* recurrences in primaquine-untreated pregnant women: an observational and modeling study in Brazil. PLoS Negl Trop Dis.

[CR16] Craig AA (1996). Molecular analysis of strains of *Plasmodium*
*vivax* from paired primary and relapse infections. J Infect Dis.

[CR17] De Niz M (2018). *Plasmodium* gametocytes display homing and vascular transmigration in the host bone marrow. Sci Adv.

[CR18] di Giovanni L (1990). On the evolutionary history of the circumsporozoite protein in plasmodia. Exp Parasitol.

[CR19] Dias S (2013). Population genetic structure of the *Plasmodium*
*vivax* circumsporozoite protein (Pvcsp) in Sri Lanka. Gene.

[CR20] Dong Y (2020). Analysis of initial laboratory diagnosis of malaria and its accuracy compared with re-testing from 2013 to 2018 in Yunnan Province. China Malar J.

[CR21] Etemadi S (2020). Genotyping and phylogenetic analysis of *Plasmodium*
*vivax* circumsporozoite protein (PvCSP) gene of clinical isolates in South-Eastern Iran. Iran J Public Health.

[CR22] Friedrich LR (2016). Complexity of infection and genetic diversity in Cambodian *Plasmodium*
*vivax*. PLoS Negl Trop Dis.

[CR23] Ghanchi NK (2019). Hematological profile and gametocyte carriage in malaria patients from Southern Pakistan. Cureus.

[CR24] Golding N (2015). Integrating vector control across diseases. BMC Med.

[CR25] Huang H (2021). The association of CYP2D6 gene polymorphisms in the full-length coding region with higher recurrence rate of vivax malaria in Yunnan Province. China Malar J.

[CR26] Imwong M (2005). Practical PCR genotyping protocols for *Plasmodium vivax* using Pvcs and Pvmsp1. Malar J.

[CR27] Imwong M (2007). Relapses of *Plasmodium vivax* infection usually result from activation of heterologous hypnozoites. J Infect Dis.

[CR28] Imwong M (2012). The first *Plasmodium vivax* relapses of life are usually genetically homologous. J Infect Dis.

[CR29] Khusmith S (1998). Antigenic disparity of *Plasmodium*
*vivax* causing initial symptoms and causing relapse. Southeast Asian J Trop Med Public Health.

[CR30] Kim JR (2006). Genetic diversity of *Plasmodium*
*vivax* in Kolkata. India Malar J.

[CR31] Kirchgatter K (1998). Molecular analysis of *Plasmodium*
*vivax* relapses using the MSP1 molecule as a genetic marker. J Infect Dis.

[CR32] Koepfli C (2009). Evaluation of *Plasmodium*
*vivax* genotyping markers for molecular monitoring in clinical trials. J Infect Dis.

[CR33] Kosaisavee V (2011). The genetic polymorphism of *Plasmodium*
*vivax* genes in endemic regions of Thailand. Asian Pac J Trop Med.

[CR34] Li XH (2021). Seven decades towards malaria elimination in Yunnan. China Malar J.

[CR35] Lin JT (2015). Using amplicon deep sequencing to detect genetic signatures of *Plasmodium*
*vivax* relapse. J Infect Dis.

[CR36] Llanos-Cuentas A (2014). Tafenoquine plus chloroquine for the treatment and relapse prevention of *Plasmodium*
*vivax* malaria (DETECTIVE): a multicentre, double-blind, randomised, phase 2b dose-selection study. Lancet.

[CR37] Looareesuwan S (1987). High rate of *Plasmodium*
*vivax* relapse following treatment of falciparum malaria in Thailand. Lancet.

[CR38] McConkey GA (1990). The generation of genetic diversity in malaria parasites. Annu Rev Microbiol.

[CR39] Nair S (2014). Single-cell genomics for dissection of complex malaria infections. Genome Res.

[CR40] NCBI (2022) https://www.ncbi.nlm.nih.gov/genome/gdv/browser/gene/?id=5472322

[CR41] Obaldia N (2018). Bone marrow is a major parasite reservoir in *Plasmodium*
*vivax* infection. mBio.

[CR42] Orjuela-Sánchez P (2009). Recurrent parasitemias and population dynamics of Plasmodium vivax polymorphisms in rural Amazonia. Am J Trop Med Hyg.

[CR43] Popovici J (2018). Genomic analyses reveal the common occurrence and complexity of *Plasmodium*
*vivax* relapses in Cambodia. mBio.

[CR44] Qari SH (1993). Identification of *Plasmodium*
*vivax*-like human malaria parasite. Lancet.

[CR45] Rosenberg R (1989). Circumsporozoite protein heterogeneity in the human malaria parasite *Plasmodium*
*vivax*. Science.

[CR46] Silva-Filho JL (2020). *Plasmodium*
*vivax* in hematopoietic niches: hidden and dangerous. Trends Parasitol.

[CR47] Sutton PL (2009). *Plasmodium*
*falciparum* and *Plasmodium*
*vivax* infections in the Peruvian Amazon: propagation of complex, multiple allele-type infections without super-infection. Am J Trop Med Hyg.

[CR48] Thanapongpichat S (2013). Microsatellite genotyping of *Plasmodium*
*vivax* infections and their relapses in pregnant and non-pregnant patients on the Thai-Myanmar border. Malar J.

[CR49] Tripathi V (2011). Evolutionary analysis of circumsporozoite surface protein and merozoite surface protein-1 (CSP and MSP-1) sequences of malaria parasites. Bioinformation.

[CR50] Võ TC (2020). Genetic polymorphism and natural selection of circumsporozoite protein in Myanmar *Plasmodium*
*vivax*. Malar J.

[CR51] White NJ (2012). Relapse. Adv Parasitol.

[CR52] White MT (2016). Variation in relapse frequency and the transmission potential of *Plasmodium*
*vivax* malaria. Proc Biol Sci.

