# Supplementary material for: Molecular identification of vivax malaria relapse patients in the Yunnan Province based on homology analysis of the Plasmodium vivax circumsporozoite protein gene
Source: Parasitol Res. 2022 Nov 5;122(1):85–96. doi: 10.1007/s00436-022-07700-7 (PMC9816221; doi:10.1007/s00436-022-07700-7)
Supplement: Supplementary file 1 — Supplementary file1 (DOC 365 KB) [file 436_2022_7700_MOESM1_ESM.doc]

**SI 1**

| **Table 1 The details of nested PCR testing for differentiating between various *Plasmodium* species** | | | | | | |
| --- | --- | --- | --- | --- | --- | --- |
| Nested PCR | Specificity of primers | Primer name^△^ | Primer sequence^△^ | Expected PCR product (bp) | Reaction conditions | Reaction systems |
| First round | Genus | rPLU5 | 5’-CCTGTTGTTGCCTTAAACTTC-3； | 1200 | 94 ° C for 3min;94° C for 30s, 58° C for 30s, 72° C for 60s, 34 cycles; 72° C for 5min. | 25 ul reaction volume including 2.6 μl template, 14.0 μl 2 × PCR Mix hybrid system (Containing Taq enzyme), 0.7 μl upstream primer (20umol / L) and 0.7 μl downstream primers (20umol / L) |
|  |  | rPLU6 | 5’-TTAAAATTGTTGCAGTTAAAACG-3’ |  |  |  |
| Second round | *P.falciparum* | rFAL1 | 5’-TTAAACTGGTTTGGGAAAACCAAATATATT-3’ | 205 | 94 ° C for 3min;94° C for 30s, 60° C for 30s, 72° C for 60s, 34 cycles; 72° C for 5min |  |
|  |  | rFAL2 | 5’-ACACAATGAACTCAATCATGACTACCCGTC-3’ |  |  |  |
|  | *P.vivax* | rVIV1 | 5’-CGCTTCTAGCTTAATCCACATAACTGATAC-3’ | 120 |  |  |
|  |  | rVIV2 | 5’-ACTTCCAAGCCGAAGCAAAGAAAGTCCTTA-3’ |  |  |  |
|  | *P.malariae* | rMAL1 | 5’-ATAACATAGTTGTACGTTAAGAATAACCGC-3’ | 141 |  |  |
|  |  | rMAL2 | 5’-AAAATTCCCATGCATAAAAAATTATACAAA-3’ |  |  |  |
|  | *P.ovale* | rOVA1 | 5’-ATCTCTTTTGCTATTTTTTAGTATTGGAGA-3’ | 800 |  |  |
|  |  | rOVA2 | 5’-GGAAAGGACACATTAATTGTATCCTAGTG-3’ |  |  |  |
| ^△^:The primers’ name and sequence were cited from References Snounou G, Viriyakosol S, Zhu XP, Jarra W, Pinheiro L, do Rosario VE, et al. High sensitivity of detection of human malaria parasites by the use of nested polymerase chain reaction. MolBiochemParasitol. 1993;61(2):315-20. | | | | | | |

**Genetic confirmation by** **Yunnan Province Malaria Diagnosis Referent Laboratory**

All of vivax malaria cases were confirmed as mono-infection by using Snounou’s method in Yunnan Province Malaria Diagnosis Referent Laboratory. The style of result was as follows.


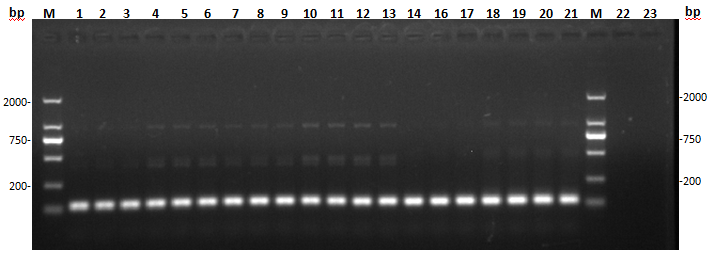


**Fig. 1** Electrophoretic image of the amplified products by nested polymerase chain reaction for 18ssRNA gene in *Plasmodium vivax* strains from vivax malaria case. （1）M: DNA Marker; (2) 22 and 23: The blank controls of first-round PCR and second-round PCR, respectively; (3) 21: *Plasmodium vivax* positive control; (4) 1-20: *Plasmodium vivax* positive samples.

| **Table 2 *Plasmodium* species result for every samples from YPMDRL** | | | | | | | |
| --- | --- | --- | --- | --- | --- | --- | --- |
| **Cases** | **ID of samples** | **The time of attack** | **Attack** | **Found cases** | **YPMDRL detection** | | |
|  |  |  |  |  | **Microscopy** | **Nest-PCR** | **Final species** |
| 1 | 69 | 2015/6/8 | primary | Tengchong, Yunnan | *P.vivax* | *P.vivax* | Mono-*P.vivax* |
|  | 70 | 2016/3/14 |  |  | *P.vivax* | *P.vivax* | Mono-*P.vivax* |
| 2 | 113 | 2015/3/4 | primary | Yingjiang, Yunnan | *P.vivax* | *P.vivax* | Mono-*P.vivax* |
|  | 114 | 2017/8/2 |  |  | *P.vivax* | *P.vivax* | Mono-*P.vivax* |
| 3 | 109 | 2015/8/31 | primary | Yingjiang, Yunnan | *P.vivax* | *P.vivax* | Mono-*P.vivax* |
|  | 110 | 2017/8/2 |  |  | *P.vivax* | *P.vivax* | Mono-*P.vivax* |
| 4 | 156 | 2016/9/9 | primary | Yingjiang, Yunnan | *P.vivax* | *P.vivax* | Mono-*P.vivax* |
|  | 157 | 2018/3/30 |  |  | *P.vivax* | *P.vivax* | Mono-*P.vivax* |
| 5 | 125 | 2016/7/5 | primary | Yingjiang, Yunnan | *P.vivax* | *P.vivax* | Mono-*P.vivax* |
|  | 126 | 2017/2/10 |  |  | *P.vivax* | *P.vivax* | Mono-*P.vivax* |
| 6 | 115 | 2016/6/3 | primary | Yingjiang, Yunnan | *P.vivax* | *P.vivax* | Mono-*P.vivax* |
|  | 116 | 2017/2/10 |  |  | *P.vivax* | *P.vivax* | Mono-*P.vivax* |
| 7 | 129 | 2014/9/9 | primary | Yingjiang, Yunnan | *P.vivax* | *P.vivax* | Mono-*P.vivax* |
|  | 130 | 2017/4/5 |  |  | *P.vivax* | *P.vivax* | Mono-*P.vivax* |
| 8 | 81 | 2015/8/31 | primary | Yingjiang, Yunnan | *P.vivax* | *P.vivax* | Mono-*P.vivax* |
|  | 82 | 2016/8/3 |  |  | *P.vivax* | *P.vivax* | Mono-*P.vivax* |
| 9 | 152 | 2016/9/9 | primary | Yingjiang, Yunnan | *P.vivax* | *P.vivax* | Mono-*P.vivax* |
|  | 153 | 2017/4/5 |  |  | *P.vivax* | *P.vivax* | Mono-*P.vivax* |
| 10 | 21 | 2016/6/16 | primary | Longchuan, Yunnan | *P.vivax* | *P.vivax* | Mono-*P.vivax* |
|  | 22 | 2017/3/21 |  |  | *P.vivax* | *P.vivax* | Mono-*P.vivax* |
| 11 | 150 | 2017/7/3 | primary | Yingjiang, Yunnan | *P.vivax* | *P.vivax* | Mono-*P.vivax* |
|  | 151 | 2018/3/26 |  |  | *P.vivax* | *P.vivax* | Mono-*P.vivax* |
| 12 | 91 | 2018/6/2 | primary | Yingjiang, Yunnan | *P.vivax* | *P.vivax* | Mono-*P.vivax* |
|  | 92 | 2018/7/15 |  |  | *P.vivax* | *P.vivax* | Mono-*P.vivax* |
| 13 | 35 | 2015/6/8 | primary | Ruili, Yunnan | *P.vivax* | *P.vivax* | Mono-*P.vivax* |
|  | 36 | 2015/8/10 |  |  | *P.vivax* | *P.vivax* | Mono-*P.vivax* |
| 14 | 145 | 2016/8/3 | primary | Yingjiang, Yunnan | *P.vivax* | *P.vivax* | Mono-*P.vivax* |
|  | 146 | 2017/5/5 |  |  | *P.vivax* | *P.vivax* | Mono-*P.vivax* |
| 15 | 19 | 2014/7/23 | primary | Longyang, Yunnan | *P.vivax* | *P.vivax* | Mono-*P.vivax* |
|  | 20 | 2015/2/2 |  |  | *P.vivax* | *P.vivax* | Mono-*P.vivax* |
| 16 | 67 | 2015/11/6 | primary | Tengchong, Yunnan | *P.vivax* | *P.vivax* | Mono-*P.vivax* |
|  | 68 | 2016/3/14 |  |  | *P.vivax* | *P.vivax* | Mono-*P.vivax* |
| 17 | 84 | 2020/1/20 | primary | Yingjiang, Yunnan | *P.vivax* | *P.vivax* | Mono-*P.vivax* |
|  | 85 | 2020/10/9 |  |  | *P.vivax* | *P.vivax* | Mono-*P.vivax* |
| 18 | 63 | 2018/5/7 | primary | Yingjiang, Yunnan | *P.vivax* | *P.vivax* | Mono-*P.vivax* |
|  | 64 | 2018/12/29 |  |  | *P.vivax* | *P.vivax* | Mono-*P.vivax* |
| 19 | 31 | 2014/7/7 | primary | Ruili, Yunnan | *P.vivax* | *P.vivax* | Mono-*P.vivax* |
|  | 32 | 2014/12/20 |  |  | *P.vivax* | *P.vivax* | Mono-*P.vivax* |
|  | 33 | 2015/4/20 |  |  | *P.vivax* | *P.vivax* | Mono-*P.vivax* |
|  | 34 | 2015/7/25 |  |  | *P.vivax* | *P.vivax* | Mono-*P.vivax* |
| 20 | 139 | 2016/7/5 | primary | Yingjiang, Yunnan | *P.vivax* | *P.vivax* | Mono-*P.vivax* |
|  | 140 | 2017/2/10 |  |  | *P.vivax* | *P.vivax* | Mono-*P.vivax* |
| 21 | 6 | 2016/5/25 | primary | Lianghe, Yunnan | *P.vivax* | *P.vivax* | Mono-*P.vivax* |
|  | 7 | 2017/5/9 |  |  | *P.vivax* | *P.vivax* | Mono-*P.vivax* |
| 22 | 59 | 2015/7/20 | primary | Yingjiang, Yunnan | *P.vivax* | *P.vivax* | Mono-*P.vivax* |
|  | 60 | 2016/4/11 |  |  | *P.vivax* | *P.vivax* | Mono-*P.vivax* |
| 23 | 23 | 2014/8/5 | primary | Yingjiang, Yunnan | *P.vivax* | *P.vivax* | Mono-*P.vivax* |
|  | 24 | 2014/10/15 |  |  | *P.vivax* | *P.vivax* | Mono-*P.vivax* |
| 24 | 4 | 2014/4/10 | primary | Guandu, Yunnan | *P.vivax* | *P.vivax* | Mono-*P.vivax* |
|  | 5 | 2014/6/3 |  |  | *P.vivax* | *P.vivax* | Mono-*P.vivax* |
| 25 | 10 | 2015/7/9 | primary | Longyang, Yunnan | *P.vivax* | *P.vivax* | Mono-*P.vivax* |
|  | 11 | 2015/8/24 |  |  | *P.vivax* | *P.vivax* | Mono-*P.vivax* |
| 26 | 123 | 2017/9/12 | primary | Yingjiang, Yunnan | *P.vivax* | *P.vivax* | Mono-*P.vivax* |
|  | 124 | 2018/6/1 |  |  | *P.vivax* | *P.vivax* | Mono-*P.vivax* |
| 27 | 119 | 2017/6/7 | primary | Yingjiang, Yunnan | *P.vivax* | *P.vivax* | Mono-*P.vivax* |
|  | 120 | 2017/8/26 |  |  | *P.vivax* | *P.vivax* | Mono-*P.vivax* |
| 28 | 61 | 2015/5/11 | primary | Tengchong, Yunnan | *P.vivax* | *P.vivax* | Mono-*P.vivax* |
|  | 62 | 2016/8/18 |  |  | *P.vivax* | *P.vivax* | Mono-*P.vivax* |
| 29 | 158 | 2018/4/9 | primary | Longling, Yunnan | *P.vivax* | *P.vivax* | Mono-*P.vivax* |
|  | 159 | 2019/1/10 |  |  | *P.vivax* | *P.vivax* | Mono-*P.vivax* |
| 30 | 1 | 2018/6/27 | primary | Yingjiang, Yunnan | *P.vivax* | *P.vivax* | Mono-*P.vivax* |
|  | 2 | 2019/5/12 |  |  | *P.vivax* | *P.vivax* | Mono-*P.vivax* |
|  | 3 | 2019/9/9 |  |  | *P.vivax* | *P.vivax* | Mono-*P.vivax* |
| 31 | 111 | 2015/6/20 | primary | Yingjiang, Yunnan | *P.vivax* | *P.vivax* | Mono-*P.vivax* |
|  | 112 | 2017/9/12 |  |  | *P.vivax* | *P.vivax* | Mono-*P.vivax* |
| 32 | 103 | 2018/6/27 | primary | Yingjiang, Yunnan | *P.vivax* | *P.vivax* | Mono-*P.vivax* |
|  | 104 | 2019/5/12 |  |  | *P.vivax* | *P.vivax* | Mono-*P.vivax* |
| 33 | 37 | 2015/7/9 | primary | Ruili, Yunnan | *P.vivax* | *P.vivax* | Mono-*P.vivax* |
|  | 38 | 2015/9/6 |  |  | *P.vivax* | *P.vivax* | Mono-*P.vivax* |
| 34 | 86 | 2019/12/22 | primary | Yingjiang, Yunnan | *P.vivax* | *P.vivax* | Mono-*P.vivax* |
|  | 87 | 2020/2/20 |  |  | *P.vivax* | *P.vivax* | Mono-*P.vivax* |
| 35 | 133 | 2017/6/1 | primary | Yingjiang, Yunnan | *P.vivax* | *P.vivax* | Mono-*P.vivax* |
|  | 134 | 2018/2/7 |  |  | *P.vivax* | *P.vivax* | Mono-*P.vivax* |
| 36 | 77 | 2018/6/19 | primary | Ruili, Yunnan | *P.vivax* | *P.vivax* | Mono-*P.vivax* |
|  | 78 | 2019/2/19 |  |  | *P.vivax* | *P.vivax* | Mono-*P.vivax* |
| 37 | 43 | 2015/8/10 | primary | Ruili, Yunnan | *P.vivax* | *P.vivax* | Mono-*P.vivax* |
|  | 44 | 2015/12/15 |  |  | *P.vivax* | *P.vivax* | Mono-*P.vivax* |
| 38 | 101 | 2016/8/20 | primary | Yingjiang, Yunnan | *P.vivax* | *P.vivax* | Mono-*P.vivax* |
|  | 102 | 2016/10/31 |  |  | *P.vivax* | *P.vivax* | Mono-*P.vivax* |
| 39 | 45 | 2014/12/30 | primary | Ruili, Yunnan | *P.vivax* | *P.vivax* | Mono-*P.vivax* |
|  | 46 | 2016/5/9 |  |  | *P.vivax* | *P.vivax* | Mono-*P.vivax* |
| 40 | 97 | 2014/8/5 | primary | Yingjiang, Yunnan | *P.vivax* | *P.vivax* | Mono-*P.vivax* |
|  | 98 | 2015/8/25 |  |  | *P.vivax* | *P.vivax* | Mono-*P.vivax* |
| 41 | 99 | 2015/7/31 | primary | Yingjiang, Yunnan | *P.vivax* | *P.vivax* | Mono-*P.vivax* |
|  | 100 | 2016/6/3 |  |  | *P.vivax* | *P.vivax* | Mono-*P.vivax* |
| 42 | 131 | 2017/7/3 | primary | Yingjiang, Yunnan | *P.vivax* | *P.vivax* | Mono-*P.vivax* |
|  | 132 | 2018/1/30 |  |  | *P.vivax* | *P.vivax* | Mono-*P.vivax* |
| 43 | 12 | 2016/1/20 | primary | Longyang, Yunnan | *P.vivax* | *P.vivax* | Mono-*P.vivax* |
|  | 13 | 2016/7/25 |  |  | *P.vivax* | *P.vivax* | Mono-*P.vivax* |
| 44 | 121 | 2016/12/29 | primary | Yingjiang, Yunnan | *P.vivax* | *P.vivax* | Mono-*P.vivax* |
|  | 122 | 2017/7/3 |  |  | *P.vivax* | *P.vivax* | Mono-*P.vivax* |
| 45 | 73 | 2014/10/11 | primary | Tengchong, Yunnan | *P.vivax* | *P.vivax* | Mono-*P.vivax* |
|  | 74 | 2015/2/5 |  |  | *P.vivax* | *P.vivax* | Mono-*P.vivax* |
| 46 | 135 | 2016/6/3 | primary | Yingjiang, Yunnan | *P.vivax* | *P.vivax* | Mono-*P.vivax* |
|  | 136 | 2017/3/7 |  |  | *P.vivax* | *P.vivax* | Mono-*P.vivax* |
| 47 | 41 | 2014/1/9 | primary | Ruili, Yunnan | *P.vivax* | *P.vivax* | Mono-*P.vivax* |
|  |  | 2014/4/9 |  |  | *P.vivax* | *P.vivax* | Mono-*P.vivax* |
| 48 | 55 | 2016/7/25 | primary | Tengchong, Yunnan | *P.vivax* | *P.vivax* | Mono-*P.vivax* |
|  | 56 | 2016/9/25 |  |  | *P.vivax* | *P.vivax* | Mono-*P.vivax* |
| 49 | 143 | 2015/4/20 | primary | Yingjiang, Yunnan | *P.vivax* | *P.vivax* | Mono-*P.vivax* |
|  | 144 | 2016/10/31 |  |  | *P.vivax* | *P.vivax* | Mono-*P.vivax* |
| 50 | 63 | 2014/11/20 | primary | Tengchong, Yunnan | *P.vivax* | *P.vivax* | Mono-*P.vivax* |
|  | 64 | 2015/3/20 |  |  | *P.vivax* | *P.vivax* | Mono-*P.vivax* |
| 51 | 8 | 2016/5/25 | primary | Longling, Yunnan | *P.vivax* | *P.vivax* | Mono-*P.vivax* |
|  | 9 | 2017/4/12 |  |  | *P.vivax* | *P.vivax* | Mono-*P.vivax* |
| 52 | 17 | 2015/7/9 | primary | Longyang, Yunnan | *P.vivax* | *P.vivax* | Mono-*P.vivax* |
|  | 18 | 2015/8/24 |  |  | *P.vivax* | *P.vivax* | Mono-*P.vivax* |
| 53 | 49 | 2014/5/20 | primary | Shidian, Yunnan | *P.vivax* | *P.vivax* | Mono-*P.vivax* |
|  | 50 | 2015/2/6 |  |  | *P.vivax* | *P.vivax* | Mono-*P.vivax* |
| 54 | 89 | 2015/8/31 | primary | Yingjiang, Yunnan | *P.vivax* | *P.vivax* | Mono-*P.vivax* |
|  | 90 | 2016/8/3 |  |  | *P.vivax* | *P.vivax* | Mono-*P.vivax* |
| 55 | 55 | 2018/8/27 | primary | Yingjiang, Yunnan | *P.vivax* | *P.vivax* | Mono-*P.vivax* |
|  | 56 | 2019/4/11 |  |  | *P.vivax* | *P.vivax* | Mono-*P.vivax* |
| 56 | 27 | 2018/5/28 | primary | Ruili, Yunnan | *P.vivax* | *P.vivax* | Mono-*P.vivax* |
|  | 28 | 2019/1/1 |  |  | *P.vivax* | *P.vivax* | Mono-*P.vivax* |
| 57 | 87 | 2015/6/20 | primary | Yingjiang, Yunnan | *P.vivax* | *P.vivax* | Mono-*P.vivax* |
|  | 88 | 2016/8/20 |  |  | *P.vivax* | *P.vivax* | Mono-*P.vivax* |
| 58 | 127 | 2015/4/20 | primary | Yingjiang, Yunnan | *P.vivax* | *P.vivax* | Mono-*P.vivax* |
|  | 128 | 2017/7/3 |  |  | *P.vivax* | *P.vivax* | Mono-*P.vivax* |
| 59 | 51 | 2019/12/28 | primary | Yingjiang, Yunnan | *P.vivax* | *P.vivax* | Mono-*P.vivax* |
|  | 52 | 2020/6/1 |  |  | *P.vivax* | *P.vivax* | Mono-*P.vivax* |
| 60 | 71 | 2015/6/8 | primary | Tengchong, Yunnan | *P.vivax* | *P.vivax* | Mono-*P.vivax* |
|  | 72 | 2016/5/5 |  |  | *P.vivax* | *P.vivax* | Mono-*P.vivax* |
| 61 | 95 | 2014/8/5 | primary | Yingjiang, Yunnan | *P.vivax* | *P.vivax* | Mono-*P.vivax* |
|  | 96 | 2015/6/20 |  |  | *P.vivax* | *P.vivax* | Mono-*P.vivax* |
| 62 | 15 | 2016/1/12 | primary | Longyang, Yunnan | *P.vivax* | *P.vivax* | Mono-*P.vivax* |
|  | 16 | 2016/3/20 |  |  | *P.vivax* | *P.vivax* | Mono-*P.vivax* |
| 63 | 154 | 2015/9/29 | primary | Yingjiang, Yunnan | *P.vivax* | *P.vivax* | Mono-*P.vivax* |
|  | 155 | 2017/6/1 |  |  | *P.vivax* | *P.vivax* | Mono-*P.vivax* |
| 64 | 79 | 2019/11/11 | primary | Yingjiang, Yunnan | *P.vivax* | *P.vivax* | Mono-*P.vivax* |
|  | 80 | 2019/12/9 |  |  | *P.vivax* | *P.vivax* | Mono-*P.vivax* |
| 65 | 147 | 2017/5/5 | primary | Yingjiang, Yunnan | *P.vivax* | *P.vivax* | Mono-*P.vivax* |
|  | 148 | 2018/6/27 |  |  | *P.vivax* | *P.vivax* | Mono-*P.vivax* |
| 66 | 47 | 2019/8/14 | primary | Yingjiang, Yunnan | *P.vivax* | *P.vivax* | Mono-*P.vivax* |
|  | 48 | 2020/4/28 |  |  | *P.vivax* | *P.vivax* | Mono-*P.vivax* |
| 67 | 57 | 2014/10/11 | primary | Tengchong, Yunnan | *P.vivax* | *P.vivax* | Mono-*P.vivax* |
|  | 58 | 2015/12/8 |  |  | *P.vivax* | *P.vivax* | Mono-*P.vivax* |
| 68 | 65 | 2019/12/25 | primary | Yingjiang, Yunnan | *P.vivax* | *P.vivax* | Mono-*P.vivax* |
|  | 66 | 2020/5/27 |  |  | *P.vivax* | *P.vivax* | Mono-*P.vivax* |
| 69 | 117 | 2017/5/5 | primary | Yingjiang, Yunnan | *P.vivax* | *P.vivax* | Mono-*P.vivax* |
|  | 118 | 2017/9/12 |  |  | *P.vivax* | *P.vivax* | Mono-*P.vivax* |
| 70 | 75 | 2017/8/21 | primary | Tengchong, Yunnan | *P.vivax* | *P.vivax* | Mono-*P.vivax* |
|  | 76 | 2017/12/14 |  |  | *P.vivax* | *P.vivax* | Mono-*P.vivax* |
| 71 | 96 | 2020/6/28 | primary | Longchuan, Yunnan | *P.vivax* | *P.vivax* | Mono-*P.vivax* |
|  | 97 | 2021/3/23 |  |  | *P.vivax* | *P.vivax* | Mono-*P.vivax* |
| 72 | 107 | 2016/7/5 | primary | Yingjiang, Yunnan | *P.vivax* | *P.vivax* | Mono-*P.vivax* |
|  | 108 | 2017/2/10 |  |  | *P.vivax* | *P.vivax* | Mono-*P.vivax* |
| 73 | 53 | 2016/4/25 | primary | Yingjiang, Yunnan | *P.vivax* | *P.vivax* | Mono-*P.vivax* |
|  | 54 | 2020/8/3 |  |  | *P.vivax* | *P.vivax* | Mono-*P.vivax* |
| 74 | 105 | 2017/3/7 | primary | Yingjiang, Yunnan | *P.vivax* | *P.vivax* | Mono-*P.vivax* |
|  | 106 | 2018/5/7 |  |  | *P.vivax* | *P.vivax* | Mono-*P.vivax* |
| 75 | 39 | 2019/5/31 | primary | Yingjiang, Yunnan | *P.vivax* | *P.vivax* | Mono-*P.vivax* |
|  | 40 | 2020/6/1 |  |  | *P.vivax* | *P.vivax* | Mono-*P.vivax* |
| 76 | 25 | 2018/6/20 | primary | Tengchong, Yunnan | *P.vivax* | *P.vivax* | Mono-*P.vivax* |
|  | 26 | 2018/8/25 |  |  | *P.vivax* | *P.vivax* | Mono-*P.vivax* |
| 77 | 29 | 2019/1/8 | primary | Yingjiang, Yunnan | *P.vivax* | *P.vivax* | Mono-*P.vivax* |
|  | 30 | 2019/8/31 |  |  | *P.vivax* | *P.vivax* | Mono-*P.vivax* |
