# Supplementary material for: Molecular identification of vivax malaria relapse patients in the Yunnan Province based on homology analysis of the Plasmodium vivax circumsporozoite protein gene
Source: Parasitol Res. 2022 Nov 5;122(1):85–96. doi: 10.1007/s00436-022-07700-7 (PMC9816221; doi:10.1007/s00436-022-07700-7)
Supplement: Supplementary file 2 — Supplementary file2 (DOC 176 KB) [file 436_2022_7700_MOESM2_ESM.doc]

**SI 2**

| **Table 1 General information records of included cases with recurrent episodes** | | | | | | | | | | | |  |
| --- | --- | --- | --- | --- | --- | --- | --- | --- | --- | --- | --- | --- |
| **Cases** | **ID of paired-**  **samples** | **Gender** | **Age** | **Found cases** | **Exposed malaria endemic areas before primary attack** | **The time of primary attack** | **Exposure history to malaria endemic areas within 30 days** | **The time of first recurrence** | **The time of second recurrence** | **The time of third recurrence** | **Interval between the first recurrence and primary attack(days)** | **Genotype of *pvcsp*** |
| 1 | 69-70 | Male | 26 | Tengchong, Yunnan | Myanmar | 2015/6/8 | No | 2016/3/14 | - | - | 280 | VK210 |
| 2 | 113-114 | Male | 30 | Yingjiang, Yunnan | Myanmar | 2015/3/4 | No | 2017/8/2 | - | - | 882 | VK210 |
| 3 | 109-110 | Male | 25 | Yingjiang, Yunnan | Myanmar | 2015/8/31 | No | 2017/8/2 | - | - | 702 | VK210 |
| 4 | 156-157 | Male | 8 | Yingjiang, Yunnan | Myanmar | 2016/9/9 | No | 2018/3/30 | - | - | 567 | VK210 |
| 5 | 125-126 | Male | 34 | Yingjiang, Yunnan | Myanmar | 2016/7/5 | No | 2017/2/10 | - | - | 220 | VK247 |
| 6 | 115-116 | Male | 36 | Yingjiang, Yunnan | Myanmar | 2016/6/3 | No | 2017/2/10 | - | - | 252 | VK210 |
| 7 | 129-130 | Female | 44 | Yingjiang, Yunnan | Myanmar | 2014/9/9 | No | 2017/4/5 | - | - | 939 | VK210 |
| 8 | 81-82 | Male | 28 | Yingjiang, Yunnan | Myanmar | 2015/8/31 | No | 2016/8/3 | - | - | 338 | VK210 |
| 9 | 152-153 | Male | 56 | Yingjiang, Yunnan | Myanmar | 2016/9/9 | No | 2017/4/5 | - | - | 208 | VK210 |
| 10 | 21-22 | Male | 29 | Longchuan, Yunnan | Myanmar | 2016/6/16 | No | 2017/3/21 | - | - | 278 | VK210 |
| 11 | 150-151 | Male | 39 | Yingjiang, Yunnan | Myanmar | 2017/7/3 | No | 2018/3/26 | - | - | 266 | VK210 |
| 12 | 91-92 | Male | 39 | Yingjiang, Yunnan | Myanmar | 2018/6/2 | No | 2018/7/15 | - | - | 43 | VK210 |
| 13 | 35-36 | Male | 44 | Ruili, Yunnan | Myanmar | 2015/6/8 | No | 2015/8/10 | - | - | 63 | VK210 |
| 14 | 145-146 | Female | 31 | Yingjiang, Yunnan | Myanmar | 2016/8/3 | No | 2017/5/5 | - | - | 275 | VK210 |
| 15 | 19-20 | Male | 63 | Longyang, Yunnan | Myanmar | 2014/7/23 | No | 2015/2/2 | - | - | 194 | VK210 |
| 16 | 67-68 | Male | 36 | Tengchong, Yunnan | Myanmar | 2015/11/6 | No | 2016/3/14 | - | - | 129 | VK210 |
| 17 | 84-85 | Male | 35 | Yingjiang, Yunnan | Myanmar | 2020/1/20 | No | 2020/10/9 | - | - | 263 | VK210 |
| 18 | 63-64 | Male | 41 | Yingjiang, Yunnan | Myanmar | 2018/5/7 | No | 2018/12/29 | - | - | 236 | VK210 |
| 19 | 31-34 | Male | 34 | Ruili, Yunnan | Myanmar | 2014/7/7 | No | 2014/12/20 | 2015/4/20 | 2015/7/25 | 166 | VK210 |
| 20 | 139-140 | Female | 25 | Yingjiang, Yunnan | Myanmar | 2016/7/5 | No | 2017/2/10 | - | - | 220 | VK210 |
| 21 | 6-7 | Male | 40 | Lianghe, Yunnan | Myanmar | 2016/5/25 | No | 2017/5/9 | - | - | 349 | VK210 |
| 22 | 59-60 | Male | 34 | Yingjiang, Yunnan | Myanmar | 2015/7/20 | No | 2016/4/11 | - | - | 266 | VK210 |
| 23 | 23-24 | Male | 31 | Yingjiang, Yunnan | Myanmar | 2014/8/5 | No | 2014/10/15 | - | - | 71 | VK210 |
| 24 | 4-5 | Male | 41 | Guandu, Yunnan | Ethiopia | 2014/4/10 | No | 2014/6/3 | - | - | 54 | VK210 |
| 25 | 10-11 | Male | 46 | Longyang, Yunnan | Myanmar | 2015/7/9 | No | 2015/8/24 | - | - | 46 | VK210 |
| 26 | 123-124 | Male | 36 | Yingjiang, Yunnan | Myanmar | 2017/9/12 | No | 2018/6/1 | - | - | 262 | VK210 |
| 27 | 119-120 | Male | 20 | Yingjiang, Yunnan | Myanmar | 2017/6/7 | No | 2017/8/26 | - | - | 80 | VK210 |
| 28 | 61-62 | Male | 41 | Tengchong, Yunnan | Myanmar | 2015/5/11 | No | 2016/8/18 | - | - | 465 | VK210 |
| 63 | 158-159 | Male | 44 | Longling, Yunnan | Myanmar | 2018/4/9 | No | 2019/1/10 | - | - | 276 | -- |
| 30 | 1-3 | Male | 28 | Yingjiang, Yunnan | Myanmar | 2018/6/27 | No | 2019/5/12 | 2019/9/9 | - | 319 | VK210 |
| 31 | 111-112 | Male | 27 | Yingjiang, Yunnan | Myanmar | 2015/6/20 | No | 2017/9/12 | - | - | 815 | VK210 |
| 32 | 103-104 | Male | 28 | Yingjiang, Yunnan | Myanmar | 2018/6/27 | No | 2019/5/12 | - | - | 319 | VK210 |
| 33 | 37-38 | Male | 43 | Ruili, Yunnan | Myanmar | 2015/7/9 | No | 2015/9/6 | - | - | 59 | VK210 |
| 34 | 86-87 | Male | 35 | Yingjiang, Yunnan | Myanmar | 2019/12/22 | No | 2020/2/20 | - | - | 60 | VK210 |
| 35 | 133-134 | Male | 45 | Yingjiang, Yunnan | Myanmar | 2017/6/1 | No | 2018/2/7 | - | - | 251 | -- |
| 36 | 77-78 | Male | 30 | Ruili, Yunnan | Myanmar | 2018/6/19 | No | 2019/2/19 | - | - | 245 | VK210 |
| 37 | 43-44 | Male | 32 | Ruili, Yunnan | Myanmar | 2015/8/10 | No | 2015/12/15 | - | - | 127 | VK210 |
| 38 | 101-102 | Male | 4 | Yingjiang, Yunnan | Myanmar | 2016/8/20 | No | 2016/10/31 | - | - | 72 | VK210 |
| 39 | 45-46 | Male | 35 | Ruili, Yunnan | Myanmar | 2014/12/30 | No | 2016/5/9 | - | - | 496 | VK210 |
| 40 | 97-98 | Male | 46 | Yingjiang, Yunnan | Myanmar | 2014/8/5 | No | 2015/8/25 | - | - | 385 | -- |
| 41 | 99-100 | Female | 52 | Yingjiang, Yunnan | Myanmar | 2015/7/31 | No | 2016/6/3 | - | - | 308 | VK210 |
| 42 | 131-132 | Female | 4 | Yingjiang, Yunnan | Myanmar | 2017/7/3 | No | 2018/1/30 | - | - | 211 | VK210 |
| 43 | 12-13 | Male | 47 | Longyang, Yunnan | Myanmar | 2016/1/20 | No | 2016/7/25 | - | - | 187 | VK210 |
| 44 | 121-122 | Female | 10 | Yingjiang, Yunnan | Myanmar | 2016/12/29 | No | 2017/7/3 | - | - | 186 | VK247 |
| 45 | 73-74 | Male | 30 | Tengchong, Yunnan | Myanmar | 2014/10/11 | No | 2015/2/5 | - | - | 117 | VK210 |
| 46 | 135-136 | Female | 10 | Yingjiang, Yunnan | Myanmar | 2016/6/3 | No | 2017/3/7 | - | - | 277 | VK210 |
| 47 | 41-42 | Male | 23 | Ruili, Yunnan | Myanmar | 2014/1/9 | No | 2014/4/9 | - | - | 90 | VK210 |
| 48 | 55-56 | Male | 28 | Tengchong, Yunnan | Myanmar | 2016/7/25 | No | 2016/9/25 | - | - | 62 | VK210 |
| 49 | 143-144 | Female | 43 | Yingjiang, Yunnan | Myanmar | 2015/4/20 | No | 2016/10/31 | - | - | 560 | VK210 |
| 50 | 63-64 | Female | 27 | Tengchong, Yunnan | Myanmar | 2014/11/20 | No | 2015/3/20 | - | - | 120 | VK210 |
| 51 | 8-9 | Male | 34 | Longling, Yunnan | Myanmar | 2016/5/25 | No | 2017/4/12 | - | - | 322 | VK210 |
| 52 | 17-18 | Male | 25 | Longyang, Yunnan | Myanmar | 2015/7/9 | No | 2015/8/24 | - | - | 46 | VK210 |
| 53 | 49-50 | Male | 51 | Shidian, Yunnan | Myanmar | 2014/5/20 | No | 2015/2/6 | - | - | 262 | VK247 |
| 54 | 89-90 | Male | 39 | Yingjiang, Yunnan | Myanmar | 2015/8/31 | No | 2016/8/3 | - | - | 338 | VK210 |
| 55 | 55-56 | Male | 38 | Yingjiang, Yunnan | Myanmar | 2018/8/27 | No | 2019/4/11 | - | - | 227 | VK210 |
| 56 | 27-28 | Male | 25 | Ruili, Yunnan | Myanmar | 2018/5/28 | No | 2019/1/1 | - | - | 218 | VK210 |
| 57 | 87-88 | Male | 29 | Yingjiang, Yunnan | Myanmar | 2015/6/20 | No | 2016/8/20 | - | - | 427 | VK210 |
| 58 | 127-128 | Female | 36 | Yingjiang, Yunnan | Myanmar | 2015/4/20 | No | 2017/7/3 | - | - | 805 | VK210 |
| 59 | 51-52 | Female | 52 | Yingjiang, Yunnan | Myanmar | 2019/12/28 | No | 2020/6/1 | - | - | 156 | VK210 |
| 60 | 71-72 | Male | 49 | Tengchong, Yunnan | Myanmar | 2015/6/8 | No | 2016/5/5 | - | - | 332 | VK210 |
| 61 | 95-96 | Male | 25 | Yingjiang, Yunnan | Myanmar | 2014/8/5 | No | 2015/6/20 | - | - | 319 | VK210 |
| 62 | 15-16 | Male | 25 | Longyang, Yunnan | Myanmar | 2016/1/12 | No | 2016/3/20 | - | - | 68 | VK210 |
| 63 | 154-155 | Male | 51 | Yingjiang, Yunnan | Myanmar | 2015/9/29 | No | 2017/6/1 | - | - | 611 | -- |
| 64 | 79-80 | Male | 23 | Yingjiang, Yunnan | Myanmar | 2019/11/11 | No | 2019/12/9 | - | - | 28 | -- |
| 65 | 147-148 | Male | 62 | Yingjiang, Yunnan | Myanmar | 2017/5/5 | No | 2018/6/27 | - | - | 418 | -- |
| 66 | 47-48 | Male | 67 | Yingjiang, Yunnan | Myanmar | 2019/8/14 | No | 2020/4/28 | - | - | 258 | -- |
| 67 | 57-58 | Male | 20 | Tengchong, Yunnan | Myanmar | 2014/10/11 | No | 2015/12/8 | - | - | 423 | -- |
| 68 | 65-66 | Male | 51 | Yingjiang, Yunnan | Myanmar | 2019/12/25 | No | 2020/5/27 | - | - | 154 | -- |
| 69 | 117-118 | Male | 3 | Yingjiang, Yunnan | Myanmar | 2017/5/5 | No | 2017/9/12 | - | - | 130 | -- |
| 70 | 75-76 | Male | 40 | Tengchong, Yunnan | Myanmar | 2017/8/21 | No | 2017/12/14 | - | - | 115 | -- |
| 71 | 196-197 | Male | 38 | Longchuan, Yunnan | Myanmar | 2020/6/28 | No | 2021/3/23 | - | - | 268 | -- |
| 72 | 107-108 | Male | 48 | Yingjiang, Yunnan | Myanmar | 2016/7/5 | No | 2017/2/10 | - | - | 220 | -- |
| 73 | 53-54 | Male | 44 | Yingjiang, Yunnan | Myanmar | 2016/4/25 | No | 2020/8/3 | - | - | 1561 | -- |
| 74 | 105-106 | Female | 37 | Yingjiang, Yunnan | Myanmar | 2017/3/7 | No | 2018/5/7 | - | - | 426 | -- |
| 75 | 39-40 | Female | 36 | Yingjiang, Yunnan | Myanmar | 2019/5/31 | No | 2020/6/1 | - | - | 367 | -- |
| 76 | 25-26 | Male | 38 | Tengchong, Yunnan | Myanmar | 2018/6/20 | No | 2018/8/25 | - | - | 66 | -- |
| 77 | 29-30 | Male | 38 | Yingjiang, Yunnan | Myanmar | 2019/1/8 | No | 2019/8/31 | - | - | 235 | -- |
