# Supplementary material for: Molecular identification of vivax malaria relapse patients in the Yunnan Province based on homology analysis of the Plasmodium vivax circumsporozoite protein gene
Source: Parasitol Res. 2022 Nov 5;122(1):85–96. doi: 10.1007/s00436-022-07700-7 (PMC9816221; doi:10.1007/s00436-022-07700-7)

**SI 3**

Sequencing peak map of *Plasmodium vivax pvcsp* gene in paired-samples from vivax malaria cases.


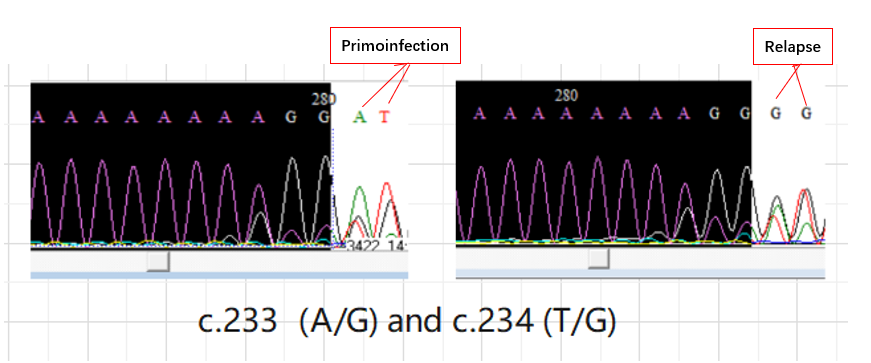


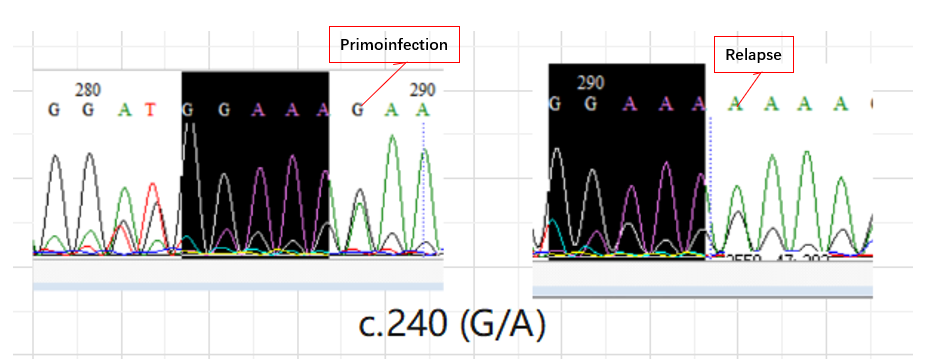


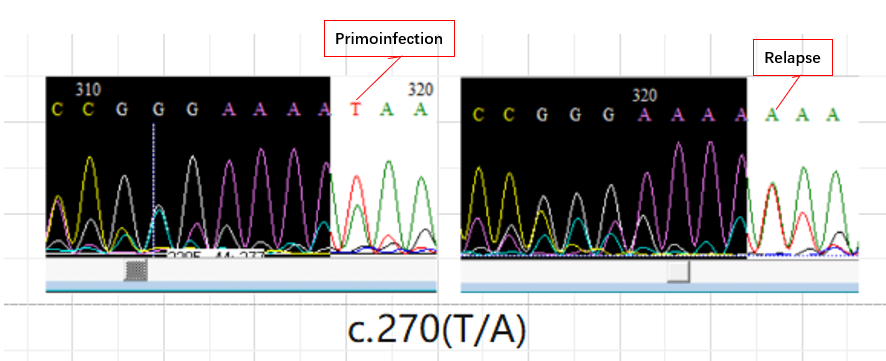


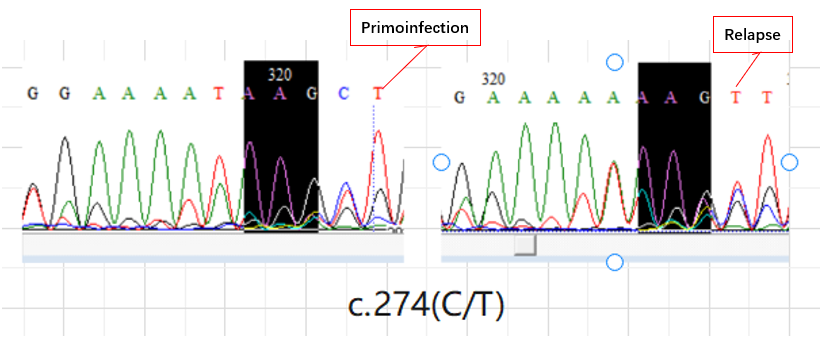


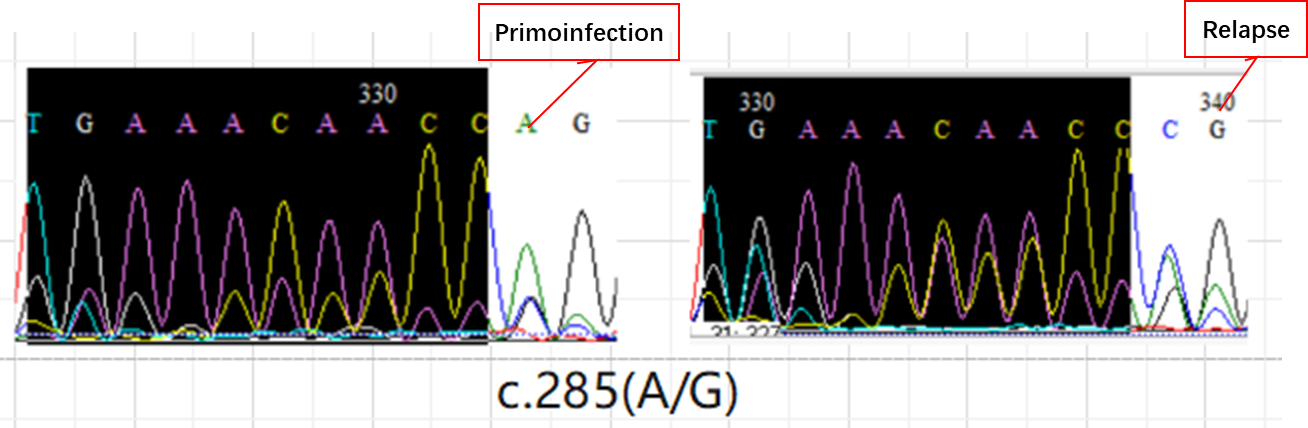


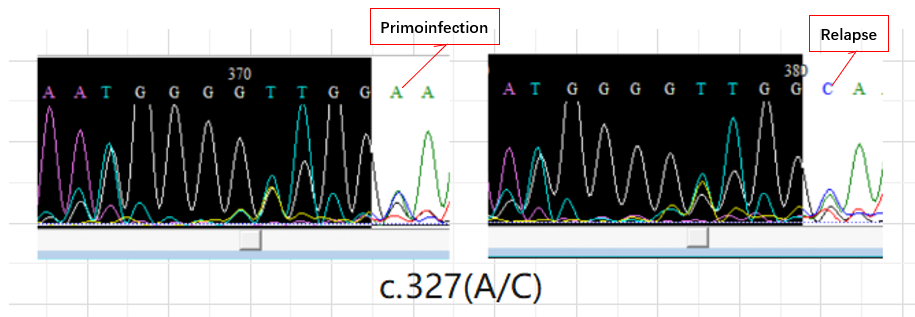


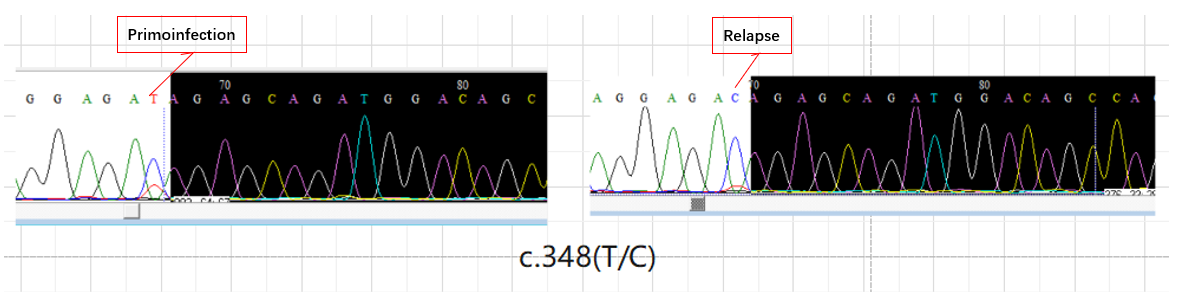


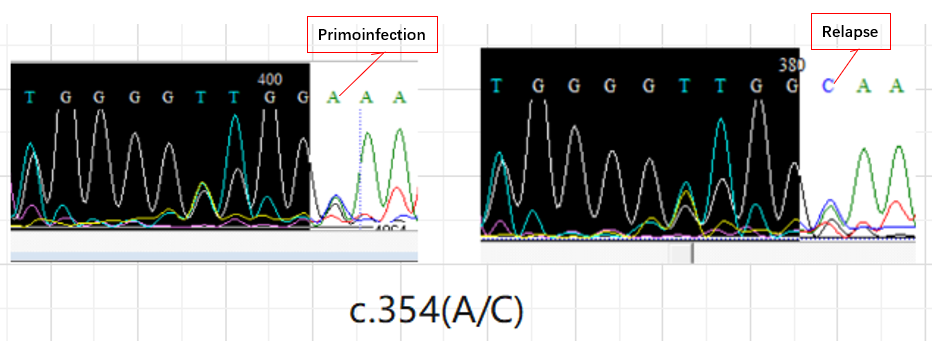


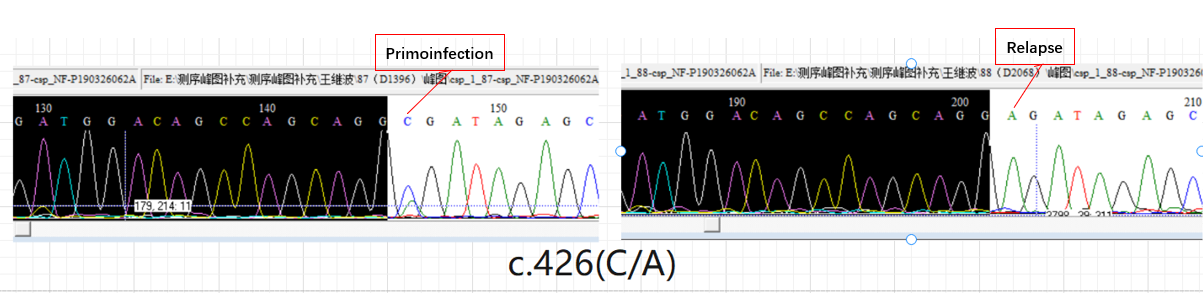


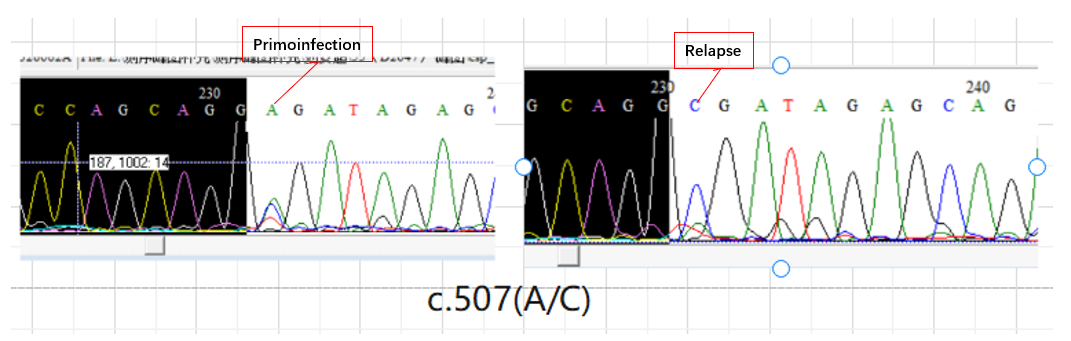


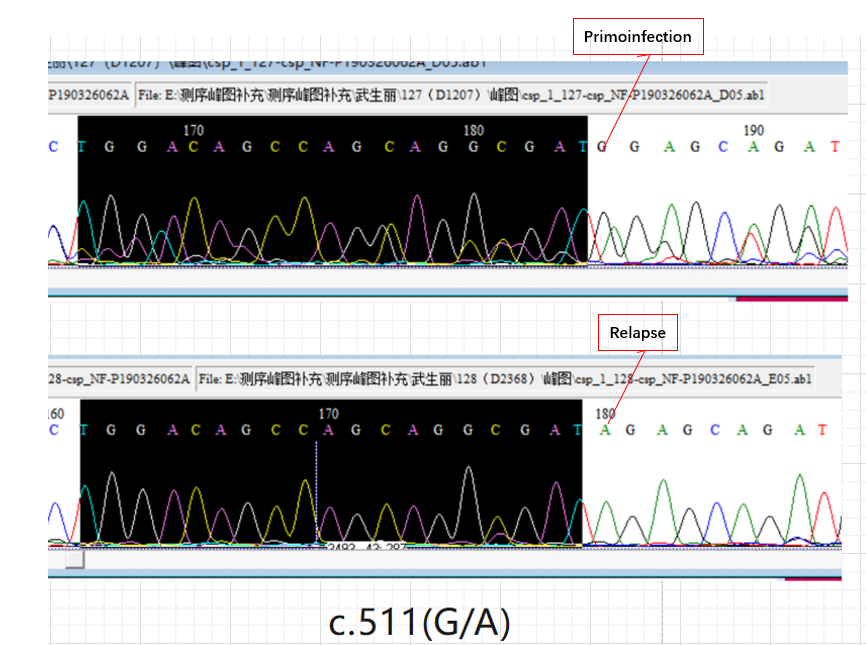


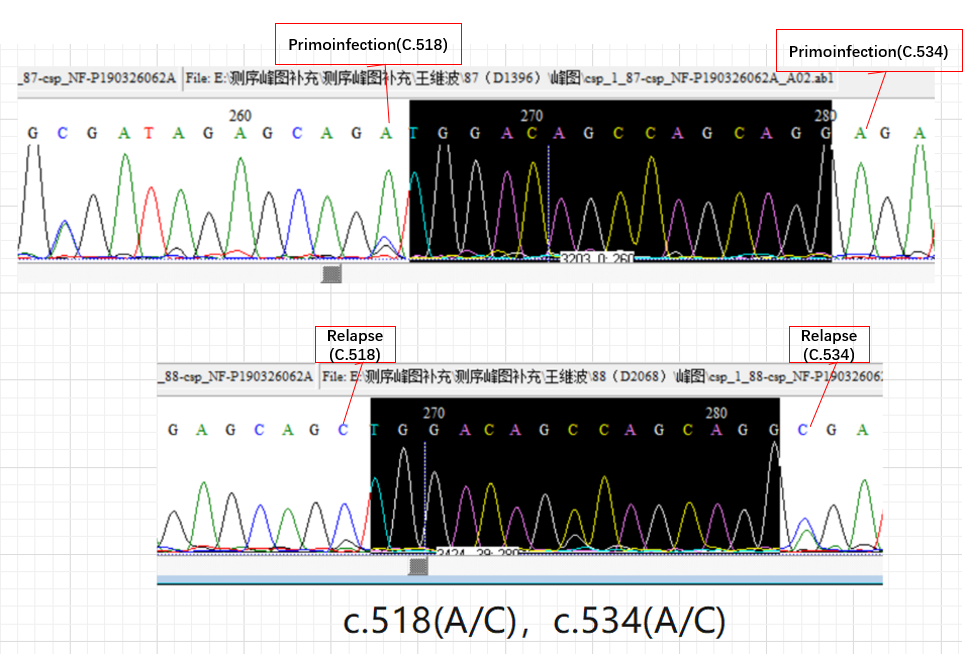


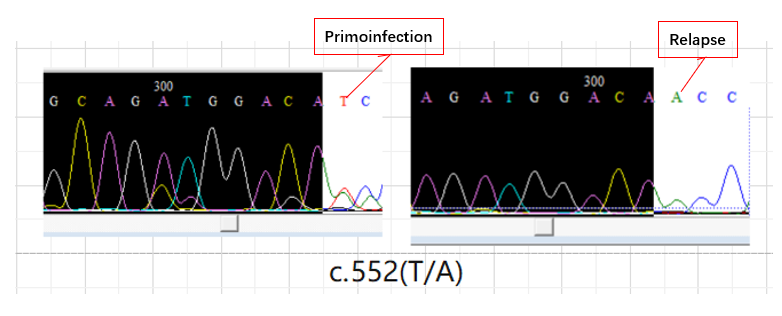


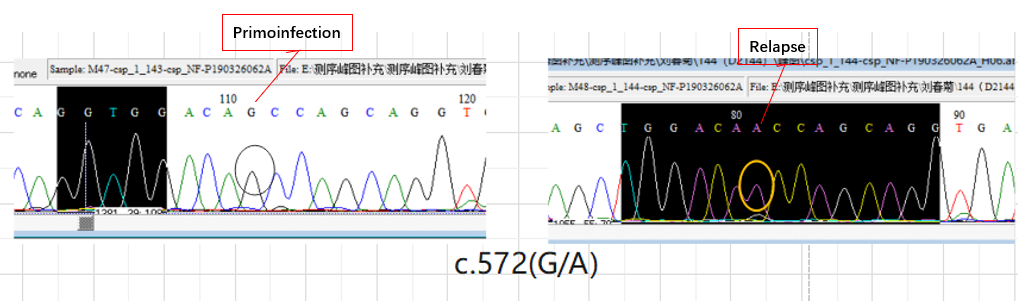


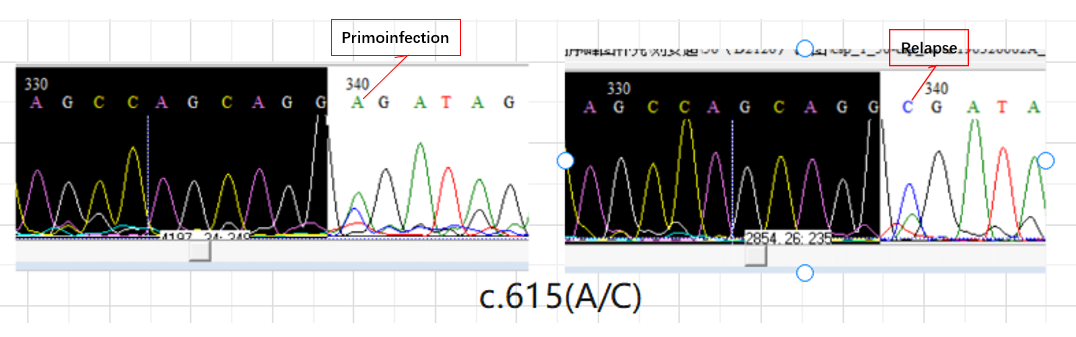


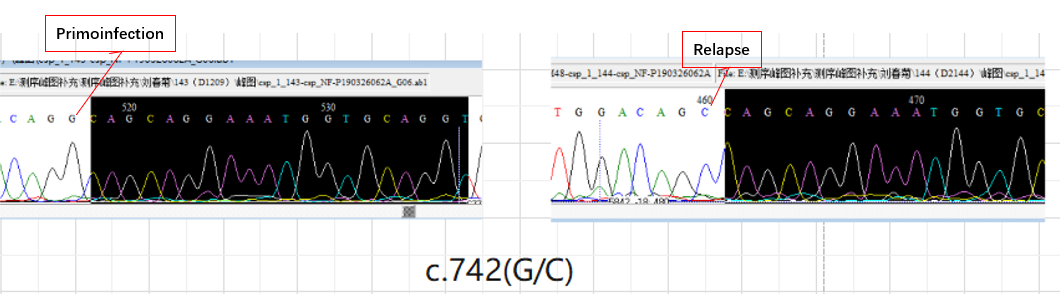


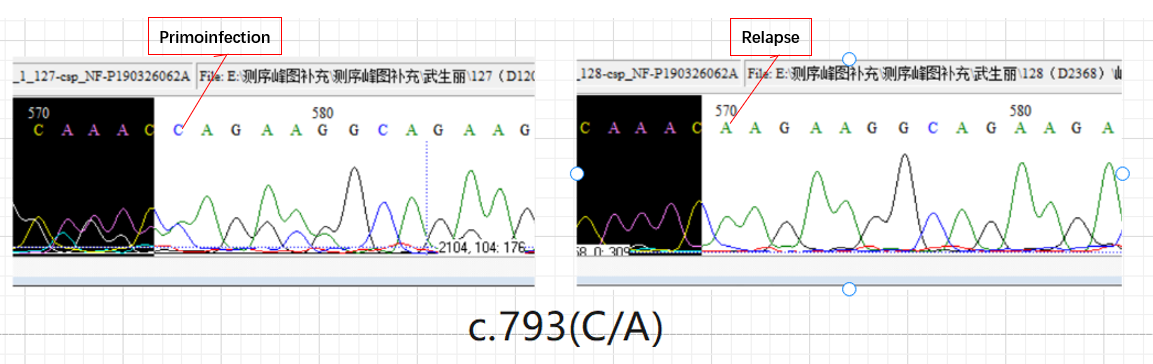


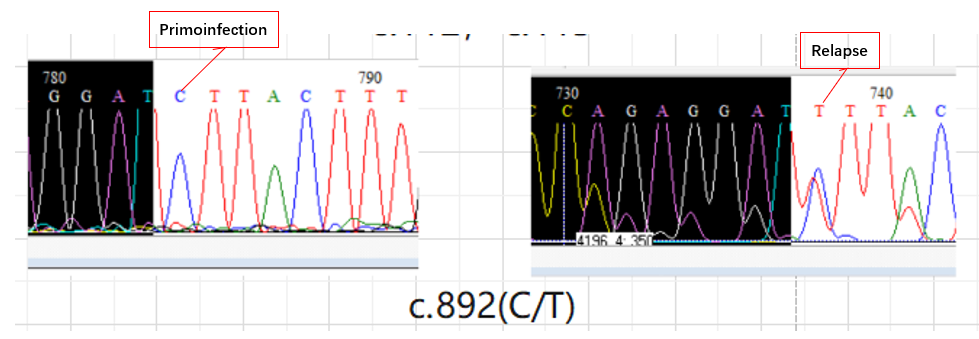

Supplement: Supplementary file 3 — Supplementary file3 (DOC 2539 KB) [file 436_2022_7700_MOESM3_ESM.doc]
