# Supplementary material for: Molecular identification of vivax malaria relapse patients in the Yunnan Province based on homology analysis of the Plasmodium vivax circumsporozoite protein gene
Source: Parasitol Res. 2022 Nov 5;122(1):85–96. doi: 10.1007/s00436-022-07700-7 (PMC9816221; doi:10.1007/s00436-022-07700-7)
Supplement: Supplementary file 4 — Supplementary file4 (DOC 603 KB) [file 436_2022_7700_MOESM4_ESM.doc]

**SI 4**

Electropherogram of the amplified products by nested PCR for *pvcsp* gene in *Plasmodium vivax* strains from some vivax malaria cases.


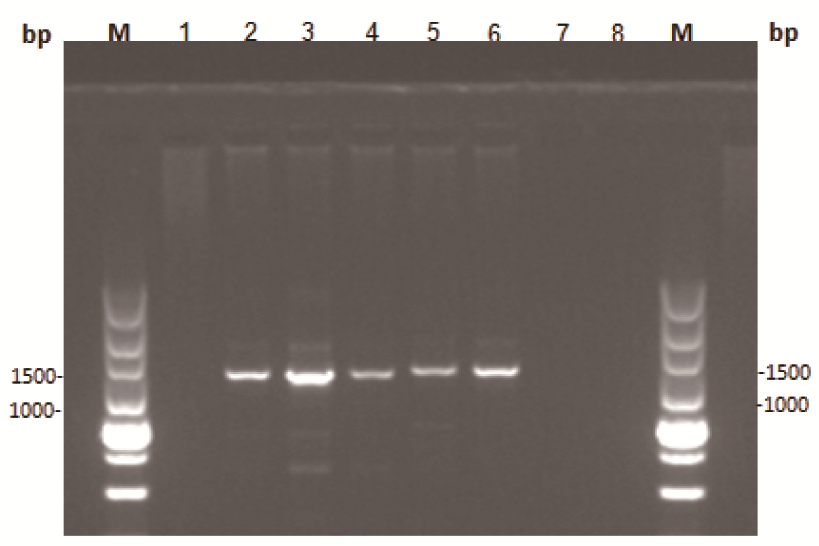


**Fig. 1 Electrophoretic image of the amplified products by nested PCR for *pvcsp* gene in *Plasmodium vivax* strains from vivax malaria cases.** (1) M: DNA Marker; (2) 7 and 8 (A): The blank controls of first-round PCR and second-round PCR, respectively; (3) 2-6: *Plasmodium vivax* positive samples; (4) 1: *Plasmodium vivax* negative samples.
