# Supplementary material for: Molecular identification of vivax malaria relapse patients in the Yunnan Province based on homology analysis of the Plasmodium vivax circumsporozoite protein gene
Source: Parasitol Res. 2022 Nov 5;122(1):85–96. doi: 10.1007/s00436-022-07700-7 (PMC9816221; doi:10.1007/s00436-022-07700-7)
Supplement: Supplementary file 5 — Supplementary file5 (DOC 69169 KB) [file 436_2022_7700_MOESM5_ESM.doc]

**SI** **5**

**Fig. 1** Alignment of amino acid sequences of completely homologous *pvcsp* genes of *Plasmodium vivax* stains from paired blood samples (Case 21)
