# Supplementary material for: Molecular identification of vivax malaria relapse patients in the Yunnan Province based on homology analysis of the Plasmodium vivax circumsporozoite protein gene
Source: Parasitol Res. 2022 Nov 5;122(1):85–96. doi: 10.1007/s00436-022-07700-7 (PMC9816221; doi:10.1007/s00436-022-07700-7)
Supplement: Supplementary file 6 — Supplementary file6 (DOC 275 KB) [file 436_2022_7700_MOESM6_ESM.doc]

**SI 6**

**The detection protocol for K10 insertion in *pvcrt-o* gene by PCR amplification**

| **Table 1 The primer protocol of PCR amplification for *pvcrt-o* gene in *Plasmodium vivax* chromosome 1** | | | | | | | |  |
| --- | --- | --- | --- | --- | --- | --- | --- | --- |
| **Fragmented amplification** | **Primer names** | **Primer sequences(5'→3')** | | **Primer**  **length (bp)** | **Product length (bp)** | **Amplification interval** | **Reference sequence of Salvador-I strain** | |
| **1** | vcrt0-1F | TTCTCCCCGCGCAGATTC | | 18 | 1459 | 330762-332220 | 1. *Plasmodium vivax* chromosome 1:NC_009906.1   (330967-334540);   1. mRNA: XM_001613407.1 2. Protein: XP_001613457.1 | |
|  | vcrt0-1R | TGAATGCGCCGACGTAATT | | 19 |  |  |  |  |
|  |  |  |  |  |  |  |  |  |
| **2** | vcrt0-5F | CAGCTGAGCATTCCGATAAACAT | | 23 | 808 | 331961-332768 |  |  |
|  | vcrt0-5R | GGACTCTCAGTAGATGCCTACTT | | 23 |  |  |  |  |
|  |  |  |  |  |  |  |  |  |
| **3** | vcrt0-9F | TGAAGCAAAGTAGGCATCTACTGAG | | 25 | 1098 | 332739-333836 |  |  |
|  | vcrt0-9R | GGAAAATCGCCGCATTAATAACCC | | 24 |  |  |  |  |
|  |  |  |  |  |  |  |  |  |
| **4** | vcrt0-11F | | TTTGAGGACTACCTGTTCAGTACT | 24 | 892 | 333787-334678 |  |  |
|  | vcrt0-11R | | CTGCGTTAAGTTTGTCAGGTGAAC | 24 |  |  |  |  |

Reference sequence (ID: NC_009906.1) (Table 1) of *Plasmodium vivax* ortholog chloroquine resistance (*pvcrt-o*) gene was used as the template to design the PCR primers and to determine the reaction condition for amplification the different target regions in *pvcrt-o* full gene. The amplification products were sequenced by Shanghai Meiji Biomedical Technology Co. Ltd, using the Sanger method.

The sequencing results were collated by using DNAStar 11.0 and BioEdit 7.2.5. The coding DNA sequence (CDS) chains of *pvcrt-o* gene from every blood sample were obtained by contig splicing. The amino acid sequence was predicted by translating CDS chain.

**The alignment of** **amino acid chains presumed from *Plasmodium vivax* chloroquine resistance transporter orthology gene**

The *pvcrt-o* genes from the *Plasmodium vivax* isolates, which were found in the paired-samples from vivax malaria cases, were amplified and sequenced. The insertion mutation of K10 was observed in *pvcrt-o* amino acid chains (figure 1), with the detection rate of 26.9% (14/52). And different haplotypes and base substitutions can be also found in CDS chains of *pvcrt-o* gene, such as c.93C>T (AGC>AG*T*), c.531A>G (AGA>AG*G*), c.811A>G (ATC>*G*TC), c.1102G>C (GCC>*C*CC), c.1120A>T (ATG>*T*TG), c.1195A>G (AAA>*G*AA) and so on, which shown synonymous and/or non-synonymous mutation at these loci (published in another paper).


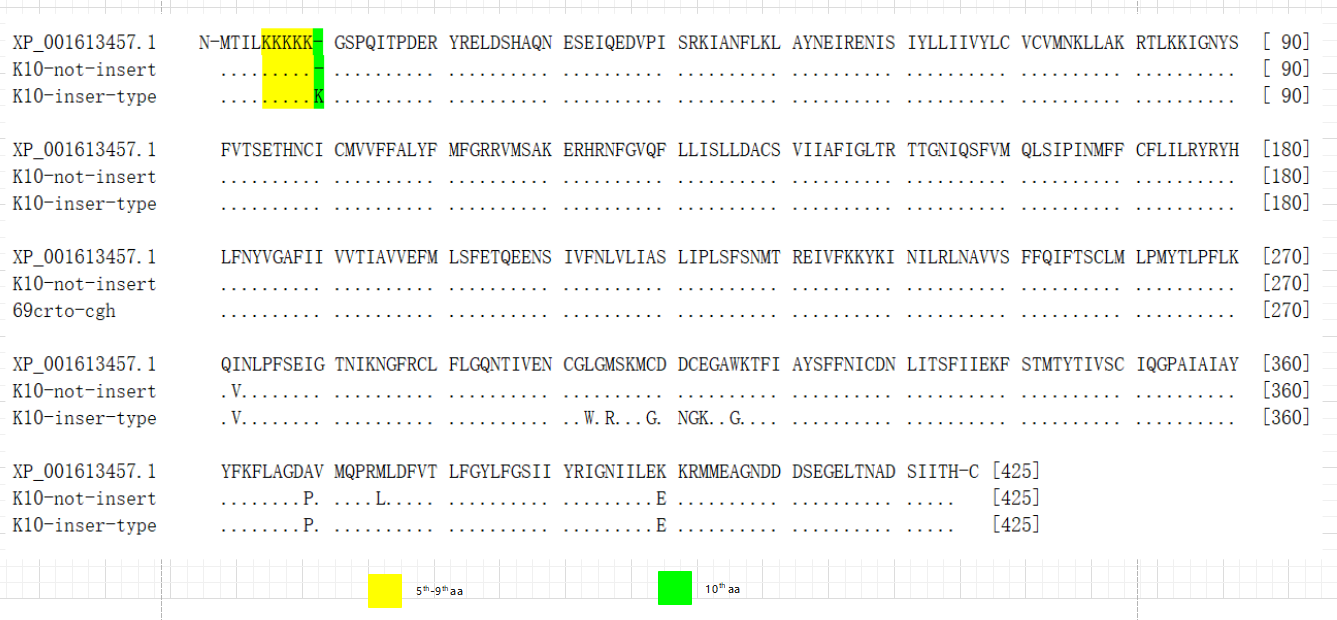


**Fig. 1** The alignment of the amino acid chains presumed from *pvcrt-o* gene in different *Plasmodium vivax* clinic strains collected from vivax malaria cases in Yunnan Province
